# Regulation of glial markers expression in the rat basolateral amygdala and hippocampus during morphine aversive memory retrieval and its extinction

**DOI:** 10.1186/s12993-025-00313-x

**Published:** 2025-12-14

**Authors:** Aurelio Franco-García, Victoria Gómez-Murcia, Cristina Núñez

**Affiliations:** 1 Group of Cellular and Molecular Pharmacology, Department of Pharmacology, CEIR Campus Mare Nostrum, University of Murcia, Murcia, 30120 Spain; 2 Instituto Murciano de Investigación Biosanitaria (IMIB)-Pascual Parrilla, Murcia, Spain; 3 The Rowett Institute, University of Aberdeen, Aberdeen, AB25 2ZD Scotland, UK

**Keywords:** Morphine, Withdrawal, CPA, Glia, Memory, Amygdala, Hippocampus

## Abstract

**Background:**

Opioid use disorder is driven by neurobehavioral adaptations where environmental cues trigger relapse. Consequently, extinction therapy (ET) aims to modify drug-associated memories but has limited long-term efficacy. Recently, evidence suggested that glial cells may contribute to neuroplasticity phenomena in addiction. In this sense, this study examined whether aversive memories of morphine withdrawal and their extinction induce transcriptional changes in glial markers (*gfap*, *aif1*, *itgam*, *klf4*) in key memory-related regions: the basolateral amygdala (BLA) and hippocampus (dentate gyrus [DG] and CA1).

**Results:**

Using the conditioned place aversion (CPA) paradigm in rats, we assessed avoidance behavior after naloxone-precipitated withdrawal and its extinction. Transcriptional analyses did not reveal major changes in the BLA. However, in CA1, downregulation of microglial markers cooccurred with aversive memory retrieval and restored after extinction. Moreover, one of the microglial markers, *klf4*, was reduced concomitantly with extinction memory retrieval in the DG. Correlation analyses showed negative associations between microglial markers and aversive memory strength, suggesting glial involvement in withdrawal-related learning.

**Conclusions:**

These findings might indicate that microglial activity in CA1 plays a role in opioid withdrawal-associated memories, and extinction training might be returning these effects to basal levels. Therefore, targeting glial responses could provide new therapeutic strategies to prevent relapse.

**Supplementary Information:**

The online version contains supplementary material available at 10.1186/s12993-025-00313-x.

## Background

Non-medical use of opioids has caused the most severe public health crisis in United States history, and it is currently expanding to European countries [1]. Complex neuro-behavioural adaptations underlie the transitioning to the addictive pathology, in which environmental associations can develop reinforcing properties and provoke relapses [2]. In this sense, not only the positive effects of drugs of abuse, but also the negative affective states developed during drug withdrawal are frequently linked with their context [3]. Although the neural basis of rewarding memory processing linked to drug consumption, and the aversive memory processing occurring during withdrawal are different[4], both associations respond to classical conditioning, and the retrieval of these memories conforms a major trigger for relapse [5]. Upon retrieval, drug-related memories are put in a labile state, where they are susceptible to be intervened [6, 7]. In this framework, extinction therapy (ET) emerged as a memory modification tool, updating the triggering element of drug-associated memories to modify the behavioral outcome [8]. Up to now, most of the work has focused on extinguishing the instrumental response for drug self-administration; however, research involving methods to eliminate associations between discrete cues and drugs of abuse is growing in clinical settings. ET has been proven to decrease the reactivity to drug cues in opioid use disorder patients [9], although it does not seem sufficient to prevent relapse overtime [10]. Thus, environmental cues can acquire long-lasting salience that enables drug-seeking behaviours [11]. Consequently, improved understanding of the molecular events in neural circuitry underlying retrieval of aversive memories and their extinction is critical to develop new psychopharmacological strategies to prevent the reinstatement of drug-related behaviours [12]. Among behavioural animal models, conditioned place aversion paradigm (CPA) is used as a highly sensitive model for measurement of the negative affective components of opiate withdrawal, which includes avoidance of cues paired with drug withdrawal negative emotional state [13–15].

Neural substrate underlying opioid use disorders is diverse, and alterations in dopaminergic activity of the reward circuitry of the brain have been extensively studied [16, 17]. Dopaminergic projections from the ventral tegmental area (VTA) towards the basolateral amygdala (BLA) and hippocampus (Hipp) are a key element in the consolidation of emotional memory associations and help integrating them with the environmental cues [18]. In this sense, dysregulation of the dopaminergic activity is a key consequence of drug of abuse consumption and participates in the development of the addicted state [19]. Literature has shown that not only neurons, but also microglia and astrocytes could participate in dopamine firing regulation since they express all the elements to synthesise, metabolise and store dopamine [20–22]. Additionally, accumulating evidence points out towards a role of glial cells in the development of neurological disorders [23, 24]. Psychostimulants like cocaine have shown to increase reactive glial state across regions [25, 26]. On the contrary, methamphetamine self-administration increased glial markers in a nucleus-dependent manner [27]. In this line, our group has previously uncovered increased astrocytic reactivity in VTA and nucleus accumbens (NAc) after morphine exposure [28, 29], and microglial modulation has also been reported [30, 31]. We have also described that extinction of aversive memories induced by morphine withdrawal is linked to decreased astrocytic levels in the infralimbic cortex (IL) and NAc, along with morphological changes in microglial cells [32]. Moreover, the recent discovery that opioids can stimulate directly glial cells [33] underlines the major potential role of these cells in the development of the addictive state.

The reactive state of glia is supported by the expression of specific markers. For astrocytic evaluation, glial fibrillary acidic protein (GFAP) is considered the hallmark to assess astrocytes reactivity and activation [34]. In microglial cells, the allograft inflammatory factor 1 (Iba1) and the alpha M subunit of the integrin Mac-1 (Cd11b/CD18) are reliable markers that have been shown to increase their expression upon inflammatory insults [35–37]. Moreover, increased levels of the krüppel-like factor 4 (klf4) in microglia has shown to mediate inflammatory responses [38].

In this work, we hypothesized that the emotional memories associated with morphine withdrawal, and their subsequent extinction, may lead to transcriptional changes in some glial markers in key memory-related brain regions. These changes could be linked to the regulation of drug-seeking behaviour, potentially offering new targets for therapeutic intervention. Consequently, we examined whether aversive memory of morphine withdrawal and/or the neutral memory after the extinction training are concurrent with changes of these markers in the Hipp (dentate gyrus -DG- and CA1) and BLA. To accomplish this, we interrogated *gfap*, *aif1*, *itgam* and *klf4* genes after morphine withdrawal aversive memory retrieval and their extinction, by using CPA paradigm in rats. Additionally, to test whether transcriptional changes could also be due to pharmacological treatment alone, a group of animals received the same drugs but did not follow conditioning procedures. Then, to try to find new potential molecular markers for behavioural outcomes, by means of correlation analysis, we analysed if some parameters were related to the transcriptional levels of the aforementioned genes.

## Methods

### Animals

Wistar male adult rats (220–240 g at the beginning of the experiment) were housed in methacrylate cages (length: 45 cm; width: 24 cm; height: 20 cm; 2–3 rats per cage) under a 12 h light/dark cycle (light: 8:00–20:00 h) in a room with controlled temperature (22 ± 2 °C). Food and water were available *ad libitum*. Animals were conditioned and tested during the light phase of the cycle. They were handled daily during the first week after arrival to minimise stress. All surgical and experimental procedures were performed in accordance with the European Communities Council Directive of 22 September 2010 (2010/63/UE) and were approved by the local Committees for animal research (Comité de Ética y Experimentación Animal; CEEA; RD 53/2013; REGA ES300305440012).

### Drugs

Morphine base was obtained from Alcaliber Laboratories (Madrid, Spain). Morphine was administered as pellets of sustained release containing morphine base (75 mg), Avicel (55 mg), polyvinylpyrrolidone (20 mg), Aerosil (0.75 mg) and magnesium stearate (1.5 mg). Placebo pellets contained the same compounds, but morphine base was replaced with lactose. Naloxone hydrochloride was purchased from Sigma Chemical (St. Louis, MO, USA), dissolved in sterile saline (0.9% NaCl; ERN Laboratories, Barcelona, Spain) and administered subcutaneously (s. c.). The dose of naloxone was 15 µg/kg and was injected in volumes of 1 ml/kg of body weight. This dose was selected given that it has been reported to evoke aversive emotional symptoms of opioid withdrawal and, consequently, elicit significant place aversion in morphine dependent animals but not in controls, and reduced physical ones [39, 40].

### Behavioural procedures

#### Induction of morphine dependence

Morphine dependence was induced by subcutaneous (s.c.) implantation of 2 morphine pellets in the interscapular area of the animals under isoflurane anaesthesia (5% for anaesthetic induction and at 2% for anaesthetic maintenance). This method has been proven to induce dependence within the next 24 h following the surgical procedure and to maintain stable the plasmatic levels of morphine for 15 days [41, 42]. Rats were randomly divided into two groups: one of them had lactose pellets implanted, and the other group was surgically intervened with morphine pellets.

Prior to surgery for the s.c. implantation of pellets, the animals were shaved at the incision site (interscapular region), and the area was subsequently disinfected with 70% alcohol. After the wound was closed with a 12-mm staple, povidone-iodine was applied to prevent possible infections.

The wound was closed with a single staple and was monitored daily and treated with antiseptics (povidone-iodine) to prevent infection. Upon awakening, both morphine-treated animals and controls showed no signs of pain. The surgical wound closed within approximately 4 days. Animals did not experience pain following surgery; therefore, no analgesia was required.

#### Conditioning apparatus

Conditioning apparatus (Panlab, Barcelona, Spain) consisted of a box separated in two same-size chambers (40 × 13 × 45 cm) connected through a rectangular corridor (25 × 13 × 45 cm). Both chambers show different visual patterns on the walls (black dots or grey stripes), different colour and texture of the floor (black or grey, smooth, or rough, respectively). The combinations were: (A) black-dotted walls, smooth black floor; and (B) black-stripped walls, rough grey floor. Walls in the corridor were transparent, which minimized the time that the animals stay in it. The position of the animal during the test and the number of entries in every chamber were detected through transduction technology and the program PPCWIN (Panlab). Experimental protocol consisted of three phases: pre-conditioning, conditioning, and test. Since chronic morphine treatment reduces weight gain because of a lower caloric intake [41, 43, 44], animal weight was measured at the same time every morning to check that morphine was properly released from the pellets.

#### Conditioning place aversion protocol (CPA)

**Fig. 1:**
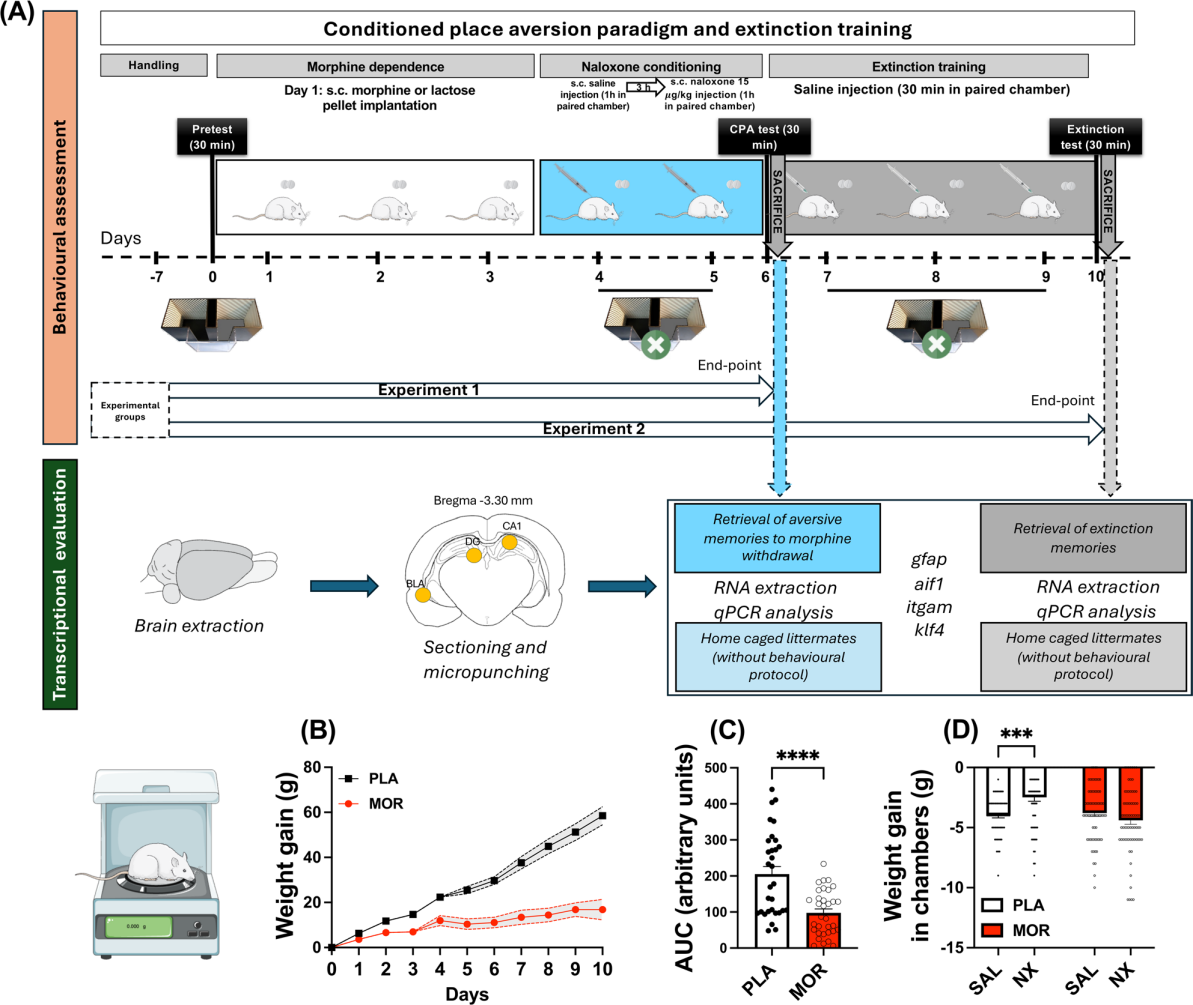
**A** Schematic representation of experimental procedure and further processing. After seven days of handling, on day 0 male wistar rats were placed in the central corridor and allowed to explore the apparatus freely for 30 min (pretest). On day 1, rats were implanted with 2 placebo or morphine pellets and were let to recover for 3 days. On day 4, in one group of animals, one chamber was randomly chosen to be paired with naloxone and the other chamber with saline (conditioning sessions) for each rat. In the group of home caged rats, same drugs were administered, but animals were returned to their cage after the injection. CPA test was conducted on day 6, exactly as in the preconditioning phase, and a set of animals were sacrificed, along with their home caged counterparts. After the test, and for 3 days, another set of animals was injected with saline. Then, a group of behaviourally challenged animals were confined in both chambers, whereas their home caged counterparts were returned to their home cage. On day 10, rats following the behavioural protocol were tested as in CPA test (extinction test) and all of them were then sacrificed. Brain was rapidly extracted, punches of the BLA, DG and CA1 were taken, and glial markers were analysed. During the experimental procedure, morphine dependent animals gained less weight over time than controls (**B**, **C**). Weight loss experienced in each conditioning chamber was the same in morphine dependent animals (**D**). *N* = 31–35 animals per group.

Briefly, CPA protocol consisted of three phases (Fig. 1A). Firstly, during the pre-conditioning phase, rats were allowed to explore freely the conditioning apparatus to test and exclude those with natural preference to any chamber. Secondly, in the conditioning phase, morphine withdrawal was induced by administration of naloxone and animals were confined to one of the compartments, which allowed them to associate the negative symptoms of withdrawal with that environment. In the last stage, animals were again allowed to freely explore the apparatus and to test whether they retrieved the environmental memories associated with the abstinence syndrome and, therefore, avoided the withdrawal-paired chamber.

Alongside these behavioural studies, and to test whether changes were due to the retrieval of memories rather than drug treatment itself, a set of animals were home caged and given the same pharmacological treatment, without exposure to the conditioning chamber at any time.

#### Pre-conditioning phase

In this phase (day 0), animals that underwent behavioural studies were placed in the central corridor and were free to explore the apparatus for 30 min (pre-test). Animals that showed natural preference or aversion for one of the chambers (more than 60% of the time and less of the 40% of the time of the session, respectively) were discarded (7 rats out of a total of 73). One chamber was randomly chosen for the animal to associate it with withdrawal syndrome to morphine, and the other was where the animal was placed after saline administration (Fig. 1A).

#### Conditioning phase

In this phase, guillotine doors blocked access from both compartments to the central corridor. Three days after pellets implantation, animals received a s.c. injection of saline and were confined in their previously assigned chamber for 1 h. Three hours after the saline administration, rats received a dose of naloxone s.c. to provoke an emotional withdrawal syndrome and were placed in the withdrawal syndrome opposite compartment for 1 h. This process was repeated for 2 consecutive days for control and morphine-treated rats. In parallel, home caged controls received the same dose of saline and naloxone but were not exposed to the conditioning box (Fig. 1A).

#### CPA test

CPA test was performed the following day after the last conditioning session, similarly to the pre-conditioning phase: animals had 30 min to explore freely both chambers. Sixty min after the CPA test started, part of the morphine-dependent animals (morphine-CPA; MOR-CPA; *N* = 11) and part of the controls (placebo-CPA; PLA-CPA; *N* = 12) were sacrificed by decapitation. At the same time, some home caged controls were also decapitated (PLA-Day 6; *N* = 3; MOR-Day 6; *N* = 4). This cohort conformed animals belonging to Experiment 1. Preference score for permanence was calculated as in Myers and Carlezon [45], in which time in saline-paired compartment was subtracted from time in naloxone-paired chamber during CPA test. Then, from this resulting score, the equally calculated score during pretest was subtracted. Preference score for entries was calculated as time change score, but regarding the number of entries to each compartment. Times and entries to each compartment alone were also analysed.

### Extinction of the CPA protocol

#### Extinction training phase

In this phase, guillotine doors blocked access from both chambers to the central corridor. After CPA testing, remaining morphine dependent and control rats followed the extinction conditioning protocol of Myers et al. [39] with several modifications. The next day after CPA test, rats were injected with saline and placed in the chamber previously assigned to saline for 30 min. After this period, rats were put back to their cages. Three h after the first injection, rats were injected again with saline and placed in the opposite chamber, previously associated with withdrawal syndrome, for 30 min. This process was repeated for 3 days. As in the conditioning phase, the remaining home caged animals received saline at the same time but were not exposed to the conditioning box.

#### Extinction of the CPA test

Extinction test was carried out similarly to the pre-conditioning and CPA tests. Control (Placebo-Extinction; PLA-EXT; *N* = 19) and morphine-treated (Morphine-Extinction; MOR-EXT; *N* = 24) rats were free to explore both compartments for 30 min. Sixty min after starting the test, animals were sacrificed through decapitation, along with their home caged controls (PLA-Day 10; *N* = 6; MOR-Day 10; *N* = 6). These animals configured Experiment 2. Then, preference scores were calculated as during the CPA test.

### RNA extraction and quantitative real-time PCR (RT-qPCR)

After behavioural procedures, rats were decapitated, brains were rapidly removed and stored immediately at −80 °C until use for quantitative real-time PCR. Four to six animals per experimental group were used in these experiments. One punch from each nucleus (BLA, DG and CA1) was extracted and homogenized with Trizol (Thermo Fisher Scientific, Waltham, MA, USA). Total RNA was extracted with the Qiagen RNeasy Lipid Tissue Mini Kit (Qiagen). For this purpose, manufacturer’s instructions were followed. RNA concentration was measured in a Qubit Fluorimeter (Thermo Fisher Scientific). One hundred ng of RNA was used for cDNA synthesis with the High-Capacity cDNA Reverse Transcription (Applied Biosystems, Waltham, MA, USA). To avoid RNA degradation, RNAase inhibitors (Applied Biosystems) were used at a final concentration of 1.0 U/µL. Retrotranscription was performed in Veriti Thermal Cycler (Applied Biosystems) and parameters were set accordingly to the manufacturer’s optimized configuration: 10 min at 25 °C, 2 h at 37 °C and 5 min at 85 °C. qPCR primers were designed NCBI tool PrimerBlast [46]. Primers (Table 1; Integrated DNA Technologies, Leuven, Belgium) were used in qPCR with SybrGreen qPCR Fast Master Mix (Applied Biosystems). qPCR experiments were carried out in the Fast Real-Time PCR System apparatus (Applied Biosystems). Forty PCR cycles were performed according to the supplier’s guidelines: 2 min at 50 °C, 10 min at 95 °C (holding stage), 15 min at 95 °C and 1 min at 60 °C (cycling stage). DEPC-treated water combined with SybrGreen Fast Master Mix was used as internal negative control in PCR templates. Amplifications were carried out in triplicate and C_q_ variations among triplicates were not greater than 0.5 cycles. Relative expression of target genes was determined by the ΔΔCT method. *Actb* (b-actin) and *ppib* (cyclophilin b) were used as loading controls. To increase accuracy, geometric mean of Cq of reference genes were calculated and use for normalisation [47].

**Table 1:** Primers used in qPCR experiments

Gene	Accession number	Forward	Reverse
*gfap*	NM_017009	AAATTGCTGGAGGGCGAAGA	CCGCATCTCCACCGTCTTTA
*aif1*	NM_017196	GGAGCTATGAGCCAGAGCAAG	CAAACTCCATGTACTTCGTCTTGA
*itgam*	NM_012711	AGCAGGGATCATTCGCTACG	GGGTGCCCTCAATTGCAAAG
*klf4*	NM_053713	GTGCCCCGACTAACCGTTG	CGTTGAACTCCTCGGTCTCC

### Data analysis

Data were analysed using GraphPad Prism 10.0 (GraphPad Software; Boston, MA, USA) and *p*-values < 0.05 were considered statistically significant. All behavioural and molecular data passed normality test (Shapiro-Wilk or Kolmogorov-Smirnov tests). For weight measurements, the area under the curve for each group of animals (placebo or morphine) was calculated and compared through unpaired *t*-test. Differences in weight gain in each compartment right after naloxone or saline injection were compared through Two-Way ANOVA with repeated measures followed by Holm-Sidák’s *post-hoc* test. For animals undergoing Experiment 1, preference scores were compared through unpaired *t*-test, whereas the rest of behavioural data across tests were analysed through Three-Way ANOVA with repeated measures followed by Holm-Sidák’s *post-hoc* test. For animals undergoing Experiment 2, preference scores were analysed through Two-Way ANOVA with repeated measures followed by Holm-Sidák’s *post-hoc* test, whereas time and entries were analysed through Three-Way ANOVA with repeated measures followed by the same *post-hoc* test. Molecular results within tests were analysed through unpaired *t*-test, and results across tests were analysed through Two-Way ANOVA, followed by Holm-Sidák’s *post-hoc* test. Absence of outliers was determined via ROUT Method (Q = 1%). To analyse a potential relationship between molecular and behavioural data, Pearson’s correlation test was performed. All descriptive data were presented as means ± standard error of the mean (SEM). All statistical outcomes are detailed in Table S1.

## Results

To ensure that morphine was efficiently released from the pellets, animal weight was measured daily. As expected, morphine dependent animals gained less weight than placebo group after pellet implantation (*t*_(62)_ = 4.619; *P* < 0.001; Fig. 1B, C), agreeing with previous reports [41, 43, 48, 49]. Accordingly, weight gain in compartments did not reveal any differences between saline and naloxone injection regarding morphine dependent animals (MOR-Sal vs. MOR-Nx; *P* = 0.1229; Fig. 1D).

### Behavioural outcome

#### Experiment 1: induction of naloxone-precipitated withdrawal and development of avoidance behaviour

**Fig. 2:**
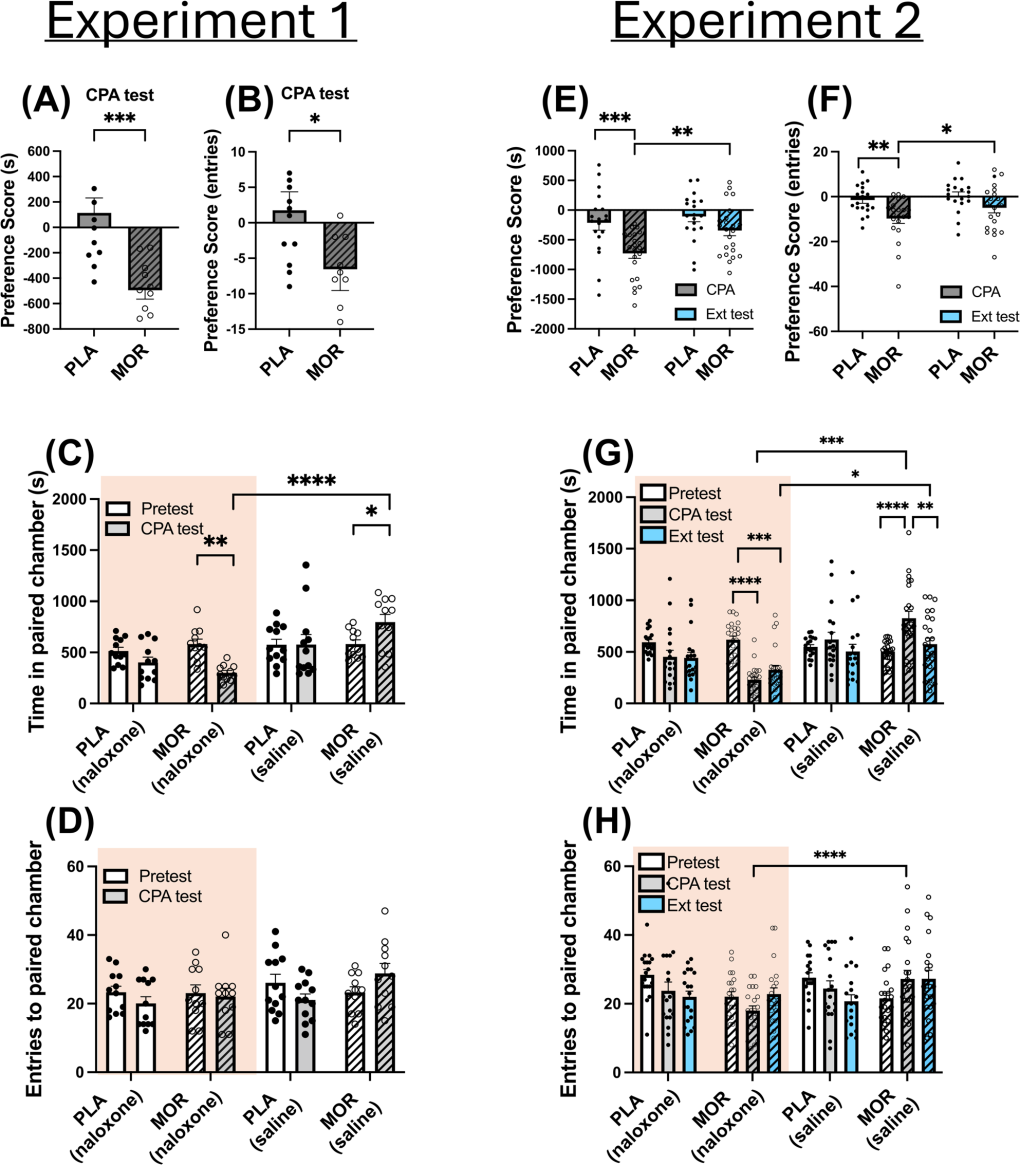
Behavioural outcome. Experiment 1: preference score of morphine-dependent animals was lower than controls both in permanence (**A**) and entries (**B**). Raw permanence in naloxone-paired chamber was diminished in placebo and morphine-dependent animals during CPA test (**C**). However, only morphine-dependent animals stayed more time in the saline-paired chamber during CPA test (**D**). In contrast, entries to naloxone-paired chamber remained unchanged across experimental groups (**E**), but entries to saline-paired chamber decreased in control group and increased in morphine-dependent animals after CPA test (**F**). Experiment 2: as in experiment 1, preference score of morphine-dependent animals was lower than controls both in permanence and entries, but they returned to control levels after three days of extinction training (**G**, **H**). Raw permanence in naloxone-paired chamber decreased during CPA test in both placebo and morphine-treated animals, and remained low after extinction test in both groups (**I**). Conversely, in morphine-dependent animals, time in saline-paired chamber increased after CPA test, and decreased after extinction training (**J**). Entries to naloxone-paired chamber decreased after Ext test in control group, but increased in morphine-dependent animals (**K**). Entries to saline-paired chamber also decreased in placebo group after Ext test, but increased in opiate-dependent animals after CPA test and remained high after Ext test (**L**). *N* = 18–24 animals per group. * *p* < 0.05, ** *p* < 0.01, *** *p* < 0.001, **** *p* < 0.0001

Behavioural results of experiment 1 showed that morphine-dependent animals developed a lower preference score towards naloxone-paired compartment compared to placebo animals, for both time (*t*_(21)_ = 4.356; *P* < 0.001) and entries (*t*_(21)_ = 2.098; *P* = 0.0482; Fig. 2A, B), thus confirming avoidance towards this chamber. Raw times revealed that morphine-dependent animals spent significantly less time in the naloxone-paired chamber than during pretest (MOR-Pretest (Nx) vs. MOR-CPA test (Nx); *P* = 0.0035) and more time in the saline-paired chamber (MOR-Pretest (Sal) vs. MOR-CPA test (Sal); *P* = 0.0375; Fig. 2C). However, entries to both naloxone and saline compartments were not significantly different in placebo and morphine-treated animals (Fig. 2D).

#### Experiment 2: suppression of avoidance behaviour through extinction training

Based on the evidence supporting that ET can reduce the impact of cues on drug-associated behaviours [50], after exhibiting CPA, a group of animals followed extinction procedures. Two-Way ANOVA revealed that morphine-dependent animals increased their preference score in both permanence (MOR-CPA test vs. MOR-Ext test; *P* = 0.0016) and entries (MOR-CPA test vs. MOR-Ext test; *P* = 0.0198) towards the naloxone-paired chamber during Ext test compared to the CPA test (Fig. 2E, F). Moreover, the lack of differences between placebo and morphine-dependent groups after ET confirmed the extinction in morphine-dependent animals.

Accordingly, in this group, raw times revealed a decrease in times spent in the naloxone-paired compartment during CPA test (MOR-Pretest (Nx) vs. MOR-CPA test (Nx); *P* < 0.0001), which tended to increase again after ET, although it did not reach statistical significance (Fig. 2G). In this same line of evidence, time in saline-paired chamber increased during CPA test but decreased during Ext test (MOR-CPA test (Sal) vs. MOR-Ext test (Sal); *P* = 0.0024; Fig. 2G). Moreover, the time morphine dependent animals spent in the saline-paired chamber was significantly higher than the time spent in the naloxone-compartment during CPA (MOR-CPA test (Nx) vs. MOR-CPA test (Sal); *P* < 0.0001) and Ext test (MOR-Ext test (Nx) vs. MOR-Ext test (Sal); *P* < 0.0366). Differences in entries were only observed between compartments in morphine-dependent animals during CPA test, revealing a higher movement towards the saline-paired environment (MOR-CPA test (Nx) vs. MOR-CPA test (Sal); *P* < 0.0001). Curiously, entries to each compartment tended to decrease after repeated testing, but they did not reach statistical significance (Fig. 2H).

### Regulation of glial markers during the retrieval of aversive and extinction memories to morphine withdrawal in the BLA and hippocampus of rats

To test whether alterations in glial markers conform a substrate for aversive memory retrieval and extinction in memory related areas, we studied by means of qPCR the transcriptional levels of *gfap* (classic marker of astrocytes), *aif1*, *itgam* (classical microglial markers) and *klf4* (found to mediate in microglial inflammation response) [38] in the BLA and the hippocampal DG and CA1.

**Fig. 3:**
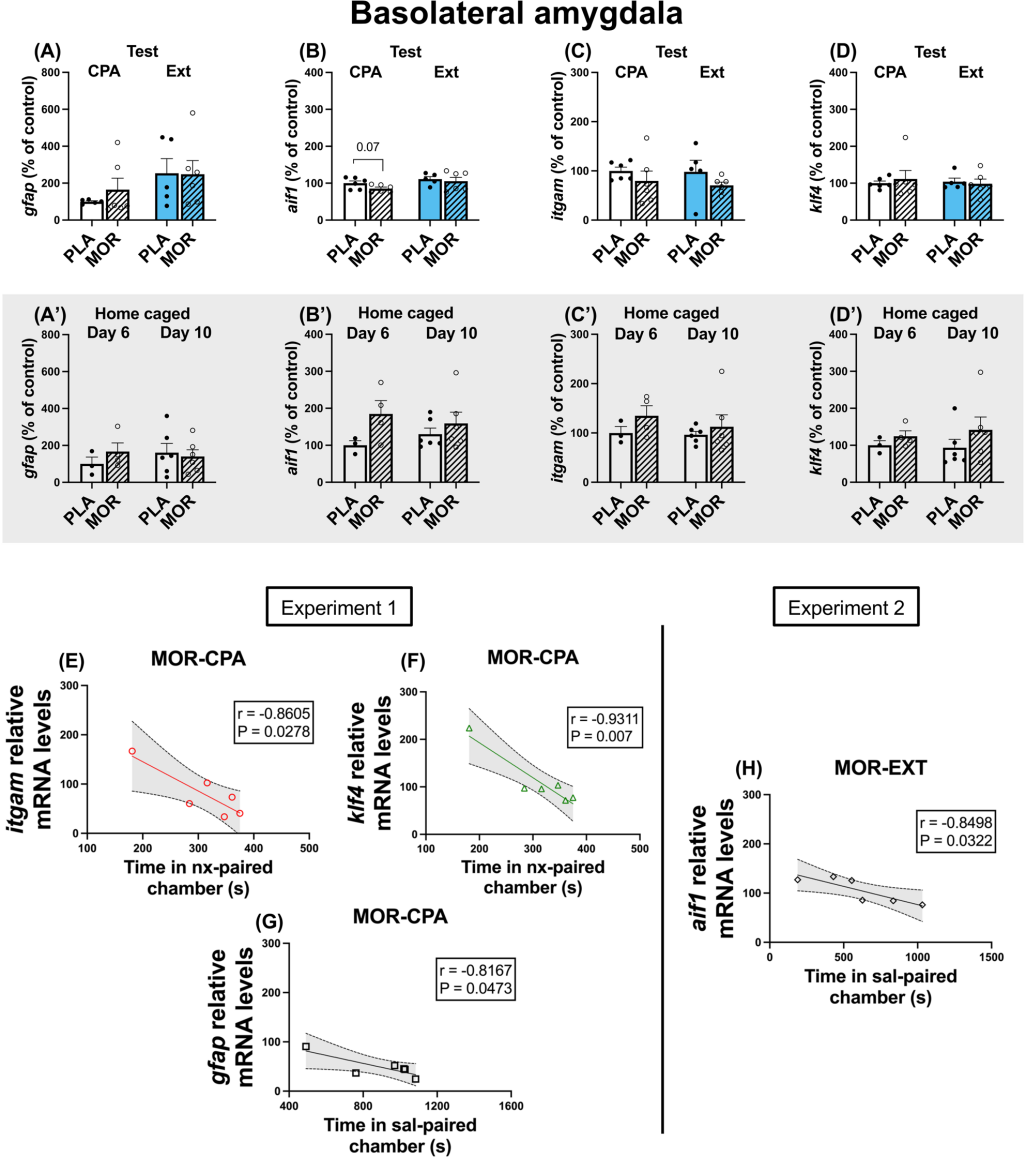
Glial markers did not change in the BLA. *Gfap* (**A**, **A’**), *aif1* (**B**, **B’**), *itgam* (**C**, **C’**) or *klf4* (**D**, **D’**) mRNA levels did not change after the retrieval of the aversive or extinction memories to morphine withdrawal in the BLA, nor in home caged controls. In experiment 1, time in naloxone-paired chambers highly correlated with *itgam* (**E**) and *klf4* (**F**) mRNA levels in morphine dependent animals during CPA test. Time in sal-paired chamber highly correlated with *gfap* mRNA levels in this same group (**G**). In experiment 2, time in sal-paired chamber during Ext test highly correlated with *aif1* mRNA levels in morphine dependent animals (H). *N* = 3–6 animals per group

In the BLA, we did not observe any changes in behaviourally assessed rats or their home caged controls regarding *gfap*, *itgam* and *klf4* (Figs. 3A, A’, C, C’, D, D’); however, we observed a trend towards diminution of *aif1* after aversive memory retrieval, which was not observed after extinction nor in home caged controls (Fig. 3B, B’). Despite the lack of changes, we observed a very high negative correlation with the time morphine-dependent animals spent in the naloxone-paired compartment and the relative *itgam* and *klf4* mRNA levels (Figs. 3E, F). Curiously, *gfap* mRNA levels also negatively correlated with the time animals spent in the saline paired chamber during CPA test (Fig. 3G), and *aif1* mRNA levels showed the same correlation after Ext test (Fig. 3H).

**Fig. 4:**
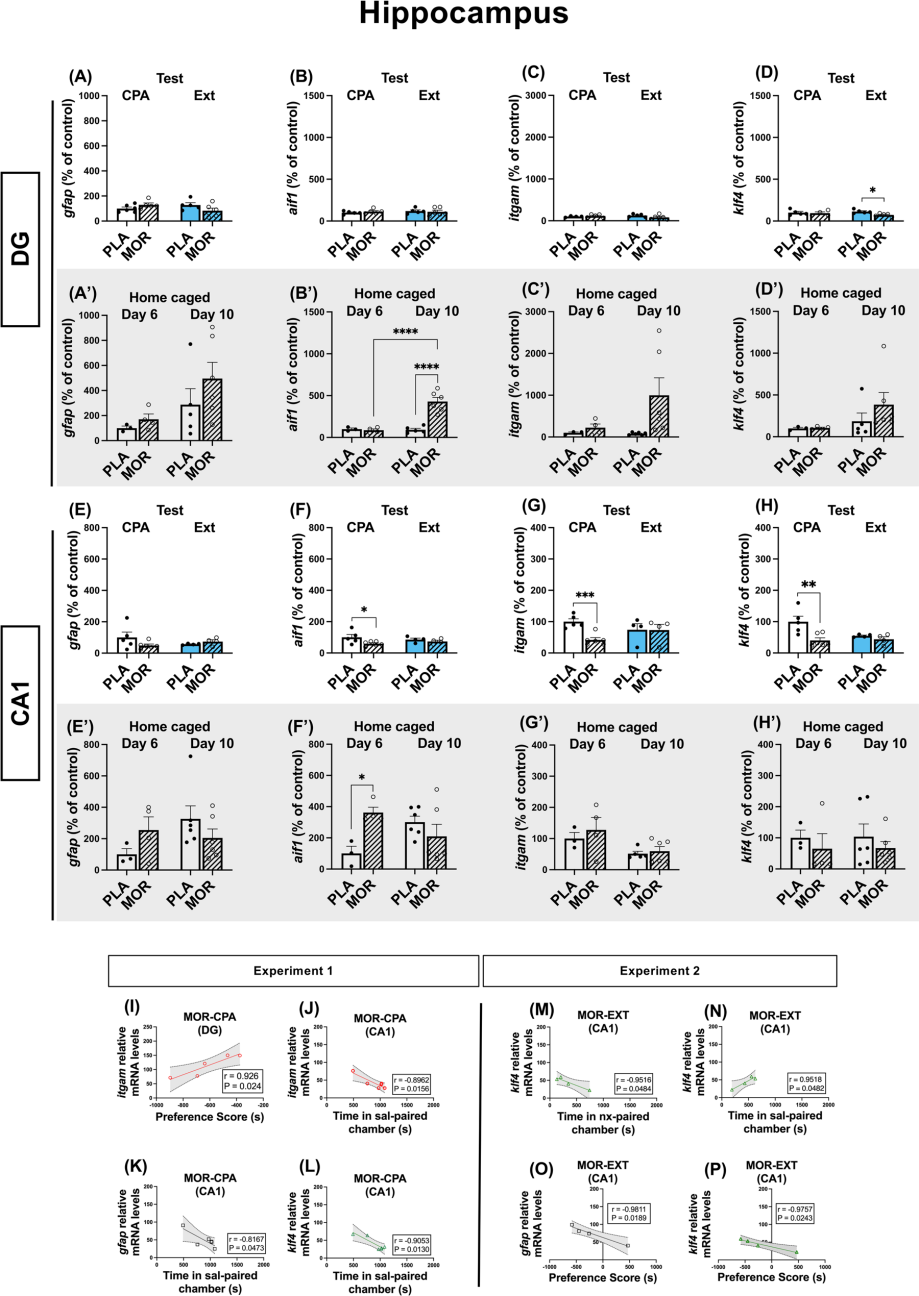
Hippocampal CA1 exhibited lower microglial markers after the retrieval of aversive memories. In the DG, nor *gfap* (**A**), *aif1* (**B**) or *itgam* (**C**) markers changed across conditions, but *klf4* was reduced in morphine-dependent animals that underwent retrieval of extinction memories (**D**). In home caged littermates, no changes were observed in *gfap* (**A’**), *itgam* (C’) or *klf4* (**D’**). However, *aif1* was increased after 10 days of morphine exposure (**B’**). In the CA1, although *gfap* mRNA levels did not exhibit any changes, the rest microglial markers were downregulated in association with the retrieval of aversive memories (**F**, **G**, **H**). In their home caged counterparts, no changes were detected in *gfap* (**E’**), *itgam* (**G’**) or *klf4* (**H’**), but *aif1* was upregulated after 6 days of morphine exposure (**F’**). In experiment 1, *itgam* mRNA levels correlated with the change score in the DG (**I**) and with the time in sal-paired chamber in the CA1 (**J**) in morphine dependent animals. *Gfap* (**K**) and *klf4* (**L**) mRNA levels in the CA1 correlated with the time animals spent in the sal-paired chamber in morphine dependent animals. In experiment 2, *klf4* mRNA levels negatively correlated with the time animals spent in the nx-paired chamber (**M**) and positively with the time animals spent in the sal-paired chamber in the CA1 of morphine dependent animals after extinction (**N**). Preference score correlated with *gfap* (**O**) and *klf4* (**P**) CA1 levels in these same animals. *N* = 3–6 animals per group. * *p* < 0.05, ** *p* < 0.01, *** *p* < 0.001

Data obtained from DG of rats following the behavioural protocol did not reveal any differences in *gfap*, *aif1*, *itgam* (Fig. 4A, B, C), however, a reduction in *klf4* expression was observed in animals that underwent the extinction of aversive memories (*t*_(8)_ = 3.242; P = 0.0118; Fig. 4D). In their home caged counterparts, we observed an upregulation in *aif1* of morphine dependent animals (*t*_(9)_ = 6.008; P < 0.001; Fig. 4B’), but not in the rest of the markers analysed (Fig. 4A’, C’, D’). Moreover, *itgam* mRNA levels of morphine-dependent animals negatively correlated with their change score during CPA test (Fig. 4J). Conversely, in CA1, results showed decreased levels of *aif1*, *itgam* and *klf4* after the retrieval of aversive memories, which returned to control levels after memory extinction (Fig. 4G, H, I). At the same time, CA1 extracted from their home caged littermates showed increased *aif1* after 6 days of morphine exposure (*t*_(5)_ = 4.710; *P* = 0.0053; Fig. 4F’), but not in *gfap* (Fig. 4E’), *itgam* (Fig. 4G’) or *klf4* (Fig. 4H’). Curiously, microglial markers levels (*itgam* and *klf4*) highly correlated with the time that animals spent in the saline-paired chamber (Fig. 4K, M) and, although no differences were observed in *gfap* levels, they also responded to the same correlation pattern (Fig. 4L). Interestingly, after extinction, *klf4* levels negatively correlated with the time spent in the naloxone-paired chamber (Fig. 4N) and positively with the time spent in the saline-paired chamber (Fig. 4O). In this group, *klf4* and *gfap* levels also negatively correlated with the time preference score achieved during extinction (Fig. 4P, Q).

## Discussion

Over the years, the main lines of research have focused on the glutamatergic, dopaminergic and opioidergic signalling in brain areas altered during drug consumption [51]; however, since glial cells participate in neurotransmission, they may also undergo changes following exposure to drugs of abuse and these changes may also be modulating neuronal pathways [52, 53]. The main reason to examine the effect of recall of environmental pairings with drug-seeking behaviours raised from the fact that these associations can develop positive or negative reinforcing properties and are one of the main trigger elements of relapses in drug-addicted patients [54]. This has led to growing interest in memory modification and its underlying mechanisms. Here, in order to explore the changes upon the retrieval of aversive memories associated with morphine withdrawal and their extinction, we used the CPA paradigm to induce aversion, followed by its forced behavioural extinction through re-exposure to the conditioned environment. In parallel, to examine the potential pharmacological effect alone, a group of animals was administered with the same drugs but were not subjected to associative learning processes. Later, in all groups, we examined the levels of glial markers in areas related to memory processing.

Behavioural results revealed that morphine-dependent animals successfully developed aversion to the naloxone-paired chamber, and that this behaviour was extinguished after 3-day ET, as we have previously shown [32, 55, 56]. In both experiments, analyses of raw times revealed decreased time in naloxone-paired chamber and increased time in saline-paired one after CPA test. After extinction, although not significant, animals tended to increase their time spent in the naloxone-associated chamber, whereas they decreased the time in the saline-associated chamber. Together, these results evidence a shift in preference towards the naloxone-paired compartment after the extinction protocol was followed, as it was reported in preference scores.

First molecular observations in rats undergoing behavioural procedures did not report any influence of morphine exposure on glial markers in the BLA and DG. Moreover, morphine treatment seemed to alter microglial markers in the CA1. Further analyses revealed that the retrieval of aversive memories was accompanied by downregulation in microglial markers (*aif1*, *itgam* and *klf4*) in the CA1, suggesting an association between these processes, but not in BLA or DG. However, in the DG, *klf4* was decreased in animals that underwent ET.

On the contrary, animals that were pharmacologically challenged, but did not follow behavioural tasks, showed increased *aif1* mRNA levels after 10 days of morphine exposure in DG. This increase was also seen in CA1, but at day 6 from morphine initial exposure. The distinction observed in the pattern of glial transcription in behaviourally tested vs. home caged counterparts suggest a differential modulation of glial levels due to memory processes and exposure to the conditioned environment rather than the pharmacological effect of the drugs administered alone. Our results might contrast with those reports studying the pattern of glial activation after chronic morphine treatment, which would reflect a neuroimmune adaptation to morphine itself. Previous studies have reported that this activation seems to be dependent of the brain nuclei and the glial cell type: chronic morphine treatment activates astrocytes in the locus coeruleus, nucleus of the solitary tract [57], lateral septal nucleus [58], VTA, dentate gyrus, CA of the Hipp, and substantia nigra (SN) [29, 59, 60]. Conversely, microglial activation seems to differ, having found that NAc, Hipp and SN remained unaltered [60]. In this set of experiments, we did not observe astrocytic marker upregulation in none of the areas analysed, either in behaviourally tested or their home caged counterparts. Additionally, upregulation of microglial markers due to drugs alone was dependent of time and region analysed: whereas BLA seemed unresponsive, different areas of the hippocampus showed increased *aif1* transcript at different times. The lower expression of glial markers that we observed in behaviourally tested rats might mirror an active, memory-dependent modulation that could partially balance the pharmacological effect. The active retrieval of the naloxone-paired aversive memory may dynamically suppress this morphine-driven microglial upregulation, resulting in normalised or significantly reduced expression of *aif1*, *itgam*, and *klf4*, particularly in the hippocampal CA1. Thus, the cognitive and emotional processing associated with memory recall could actively adjust the glial activation caused by chronic morphine [29, 57–60], highlighting an interaction between memory-related circuits and opioid-driven neuroimmune changes.

The lack of glial changes observed after behavioural extinction agree with previous studies from our group: we also reported that Iba1 immunoreactivity remained unaltered after ET in several areas belonging to the mesocorticolimbic circuitry, and despite this fact, the shape of microglial cells was affected [32]. Also, the region-specific patterns found in this work might reflect the influence of repeated withdrawal, which has been shown to exert effects opposite to morphine dependence on glial gene regulation in striatum[61]. For instance, heroin abstinence induces astrocytic remodelling in the DG [62], whereas hippocampal microglia appear largely unresponsive during withdrawal [63]. Similarly, single-cell analyses in the amygdala indicate that morphine dependence and withdrawal trigger immune pathways in opposite directions[64, 65]. Nonetheless, it is important to note that the low dose of naloxone used may also cause a different molecular response compared to previous research involving forced withdrawal, in which naloxone doses go up to 1 mg/kg, and somatic signs of withdrawal are evident [66, 67]. Altogether, these findings suggest that morphine’s neuroimmune consequences are shaped not only by time-dependent drug exposure but also by the withdrawal context, which may account for our observed reductions in glial markers in behaviourally tested animals.

Besides, variability in chronic morphine administration protocols likely contributes to inconsistencies in reported glial reactivity. Whereas the rationale behind the use of escalating morphine doses relies on the fact that certain degree of tolerance may be developed over time [68], it might be also influencing the degree of insult that glial cells are receiving. In our model, and in agreement with previous reports [32, 39, 55, 56], morphine-dependent animals successfully developed aversion to the naloxone-paired compartment after CPA training, even when a minimal dose of naloxone was injected, thus endorsing that steady release of morphine into the bloodstream by pellet implantation is effective at inducing drug-seeking behaviours. Moreover, the reduction in weight gain also supports the effectiveness of pellet implantation in inducing dependence [41, 43]. However, and despite showing stable analgesic responses up to 12 days post-implantation [69], it remains uncertain whether molecular tolerance occurs at a subclinical level. In this line, it is possible that the steady release of morphine throughout 6 to 10 days could be triggering allostatic glial compensatory mechanisms to overcome sustained morphine exposure in some brain areas. These mechanisms might also be accountable for the time-dependent changes observed in home caged animals, in which increase of *aif1* mRNA levels in CA1 is seen after 6 days but not after 10 days of morphine exposure. Moreover, since naloxone was administered one day before the sacrifice, it is unlikely that the effect might be due to naloxone direct effect alone due to its rapid clearance [70]. Supporting this hypothesis, recent findings have reported that morphine tolerance is only linked to glial hyperactivation in the spinal cord, but not at the brain level, where no changes were detected [71]. However, changes observed in DG at 10 days might reflect region-specific dynamics in response to morphine.

On the other hand, the effects seen on CA1 of behaviourally tested animals, considering that naloxone was only administered during conditioning days, and the test was conducted the following day, are unlikely to be due to a direct effect of naloxone per se, but rather the recall of naloxone-induced withdrawal memories. Despite not having found significant changes after the retrieval of morphine withdrawal-associated aversive memories in the BLA and DG, several behavioural parameters highly correlated with the expression levels of glial markers, with the greatest number of significant correlations found in the aversive memory retrieval group, which supports the link between the activity of glial cells and behaviour. In the BLA, microglial markers expression highly and negatively correlated with the time animals spent in the naloxone-paired chamber. These data might reflect the relationship between the degree of aversion, and a higher microglial activation in emotional memory areas. At the same time, the time spent in the saline-paired chamber during CPA test strongly and negatively correlated with microglial levels of CA1, which also strengthen the relationship between aversive behaviour and glial levels, showing that, although total mRNA levels are diminished in this group, lower individual levels might correspond to a higher degree of aversion achieved. This could also show that a more successful avoidance of the naloxone-paired chamber -by increased time spent in the saline-paired side- might be related to decreased glial activation. Curiously, after extinction, these analyses in the CA1 revealed that increased time in the compartment previously paired with naloxone could be related to lower glial expression. Although they seem conflicting, these data could reflect a successful impairment of the environmental emotional valence and the glial levels, also supported by the absence of changes observed after extinction training in all the areas analysed.

To our knowledge, insights into the role of glia in memory processing mechanisms come from diseases that co-occur with mnemonic processes. Although Alzheimer’s disease is characterised by increased in astrocytic number surrounding amyloid plaques [72], the question regarding the effect of their intervention in memory impairments is still unanswered. In this sense, increased levels of plasmatic GFAP have been found to be correlated with poorer memory scores in patients [73]. Recent findings have reported the predominant role of glial mechanisms and glutamate release in aversive memory processing [74]. Also, manipulation of astrocytic activity in the CA1 and BLA has shown to promote the formation of contextual fear memory [75, 76]. However, the contribution of these areas’ glial activity to the maladaptive learning underlying substance use disorders remains unexplored. Our findings provide new insights into the relationship between glial markers and aversive memory retrieval associated with morphine withdrawal. While chronic morphine exposure has been linked to region-specific glial activation, our results indicate that aversive memory recall is associated with the downregulation of microglial markers selectively in the CA1, suggesting a dynamic role of glial cells in memory-related adaptations. Interestingly, the correlation between glial markers expression and behavioural parameters further supports the hypothesis that glial activity may influence the strength of aversive associations.

In this work, the observed differences in glial response across brain regions highlight the complexity of glial-neuronal interactions in substance use disorders and suggest that glial contributions to memory processing may vary depending on brain region, exposure patterns, and withdrawal states. Given the growing recognition of glial involvement in synaptic plasticity and learning, future studies should explore whether targeted modulation of glial activity could alter the persistence of drug-associated memories. Identifying the molecular mechanisms underlying these changes may provide novel therapeutic opportunities to mitigate the maladaptive learning that contributes to relapse. Ultimately, our findings underscore the need for a more comprehensive understanding of how glial cells shape memory processes in the addictive pathology, paving the way for potential interventions aimed at disrupting the pathological memory traces that sustain drug-seeking behaviours.

## Supplementary Information

Supplementary Material 1.

## Data Availability

The datasets of the current study are available from the corresponding author on reasonable request.

## References

[1] Rivera BD, Friedman SR. What would it really take to solve the overdose epidemic in the United States? Int J Drug Policy. 2024;128:104435. 38729061 10.1016/j.drugpo.2024.104435 PMC11220856

[2] Piazza PV, Deroche-Gamonet V. A multistep general theory of transition to addiction. Psychopharmacology. 2013;229:387–413. 23963530 10.1007/s00213-013-3224-4 PMC3767888

[3] Smith MA. Nonhuman animal models of substance use disorders: translational value and utility to basic science. Drug Alcohol Depend. 2020;206:107733. 31790978 10.1016/j.drugalcdep.2019.107733 PMC6980671

[4] Volkow ND, Michaelides M, Baler R. The neuroscience of drug reward and addiction. Physiol Rev. 2019;99:2115–40. 31507244 10.1152/physrev.00014.2018 PMC6890985

[5] Olive MF, Kalivas PW. Conditioning of Addiction. In: Addiction Medicine. New York, NY: Springer New York; 2010. p. 159–78. 10.1007/978-1-4419-0338-9_8.

[6] Inda MC, Muravieva EV, Alberini CM. Memory retrieval and the passage of time: from reconsolidation and strengthening to extinction. J Neurosci. 2011;31:1635–43. 21289172 10.1523/JNEUROSCI.4736-10.2011 PMC3069643

[7] Nader K, Schafe GE, Le Doux JE. Fear memories require protein synthesis in the amygdala for reconsolidation after retrieval. Nature. 2000;406:722–6. 10963596 10.1038/35021052

[8] Torregrossa MM, Taylor JR. Learning to forget: manipulating extinction and reconsolidation processes to treat addiction. Psychopharmacology. 2013;226:659–72. 22638814 10.1007/s00213-012-2750-9 PMC3466391

[9] Franken IH, de Haan HA, van der Meer CW, Haffmans PM, Hendriks VM. Cue reactivity and effects of cue exposure in abstinent posttreatment drug users. J Subst Abuse Treat. 1999;16:81–5. 9888125 10.1016/s0740-5472(98)00004-x

[10] Marissen MAE, Franken IHA, Blanken P, van den Brink W, Hendriks VM. Cue exposure therapy for the treatment of opiate addiction: results of a randomized controlled clinical trial. Psychother Psychosom. 2007;76:97–105. 17230050 10.1159/000097968

[11] Hyman SE. Addiction: A Disease of Learning and Memory. Am J Psychiatry. 2005;162:1414–22. 16055762 10.1176/appi.ajp.162.8.1414

[12] Myers KM, Carlezon WA Jr. Extinction of drug- and withdrawal-paired cues in animal models: relevance to the treatment of addiction. Neurosci Biobehav Rev. 2010;35:285–302. 20109490 10.1016/j.neubiorev.2010.01.011 PMC2990695

[13] O’Brien CP, Testa T, O’Brien TJ, Brady JP, Wells B. Conditioned narcotic withdrawal in humans. Science (1979). 1977;195:1000–2. 10.1126/science.841320 841320

[14] García-Pérez D, Ferenczi S, Kovács KJ, Laorden ML, Milanés MV, Núñez C. Glucocorticoid homeostasis in the dentate gyrus is essential for opiate withdrawal-associated memories. Mol Neurobiol. 2017;54:6523–41. 27730515 10.1007/s12035-016-0186-7

[15] Stinus L, Caille S, Koob GF. Opiate withdrawal-induced place aversion lasts for up to 16 weeks. Psychopharmacology. 2000;149:115–20. 10805605 10.1007/s002139900358

[16] Nestler EJ. Molecular basis of long-term plasticity underlying addiction. Nat Rev Neurosci. 2001;2:119–28. 11252991 10.1038/35053570

[17] Nestler EJ. Is there a common molecular pathway for addiction? Nat Neurosci. 2005;8:1445–9. 16251986 10.1038/nn1578

[18] MacNicol B. The biology of addiction. Can J Anaesth. 2017;64:141–8. 27837404 10.1007/s12630-016-0771-2

[19] Koob G. Drug addiction, dysregulation of reward, and allostasis. Neuropsychopharmacology. 2001;24:97–129. 11120394 10.1016/S0893-133X(00)00195-0

[20] Mastroeni D, Grover A, Leonard B, Joyce JN, Coleman PD, Kozik B, et al. Microglial responses to dopamine in a cell culture model of Parkinson’s disease. Neurobiol Aging. 2009;30:1805–17. 18325635 10.1016/j.neurobiolaging.2008.01.001 PMC2762863

[21] Mackie P, Lebowitz J, Saadatpour L, Nickoloff E, Gaskill P, Khoshbouei H. The dopamine transporter: an unrecognized nexus for dysfunctional peripheral immunity and signaling in Parkinson’s disease. Brain Behav Immun. 2018;70:21–35. 29551693 10.1016/j.bbi.2018.03.020 PMC5953824

[22] Matt SM, Gaskill PJ. Where is dopamine and how do immune cells see it?: Dopamine-mediated immune cell function in health and disease. J Neuroimmune Pharmacol. 2020;15:114–64. 31077015 10.1007/s11481-019-09851-4 PMC6842680

[23] Adamczyk A. Glial-neuronal interactions in neurological disorders: molecular mechanisms and potential points for intervention. Int J Mol Sci. 2023. 10.3390/ijms24076274. 37047246 10.3390/ijms24076274 PMC10094708

[24] Du L, Zhang Y, Chen Y, Zhu J, Yang Y, Zhang H-L. Role of microglia in neurological disorders and their potentials as a therapeutic target. Mol Neurobiol. 2017;54:7567–84. 27830532 10.1007/s12035-016-0245-0

[25] Liao K, Guo M, Niu F, Yang L, Callen SE, Buch S. Cocaine-mediated induction of microglial activation involves the ER stress-TLR2 axis. J Neuroinflammation. 2016;13:33. 26860188 10.1186/s12974-016-0501-2 PMC4748483

[26] Little KY, Ramssen E, Welchko R, Volberg V, Roland CJ, Cassin B. Decreased brain dopamine cell numbers in human cocaine users. Psychiatry Res. 2009;168:173–80. 19233481 10.1016/j.psychres.2008.10.034

[27] Armstrong V, Reichel CM, Doti JF, Crawford CA, McDougall SA. Repeated amphetamine treatment causes a persistent elevation of glial fibrillary acidic protein in the caudate-putamen. Eur J Pharmacol. 2004;488:111–5. 15044042 10.1016/j.ejphar.2004.02.001

[28] García-Pérez D, Laorden ML, Milanés MV. Regulation of pleiotrophin, midkine, receptor protein tyrosine phosphatase β/ζ, and their intracellular signaling cascades in the nucleus accumbens during opiate administration. Int J Neuropsychopharmacol. 2015;19:pyv077. 26164717 10.1093/ijnp/pyv077 PMC4772269

[29] García-Pérez D, Luisa Laorden M, Núñez C, Victoria Milanés M. Glial activation and midkine and pleiotrophin transcription in the ventral tegmental area are modulated by morphine administration. J Neuroimmunol. 2014;274:244–8. 25108770 10.1016/j.jneuroim.2014.07.017

[30] Yang Y, Sun Y, Hu R, Yan J, Wang Z, Li W, et al. Morphine promotes microglial activation by upregulating the EGFR/ERK signaling pathway. PLoS One. 2021. 10.1371/journal.pone.0256870. 34520454 10.1371/journal.pone.0256870 PMC8439491

[31] Nam MH, Han KS, Lee J, Won W, Koh W, Bae JY, et al. Activation of astrocytic μ-opioid receptor causes conditioned place preference. Cell Rep. 2019;28:1154-1166.e5. 31365861 10.1016/j.celrep.2019.06.071

[32] Franco‐García A, Gómez‐Murcia V, Milanés MV, Núñez C. Dopamine D _3_ receptor blockade accelerates the extinction of opioid withdrawal‐induced drug‐seeking behaviours and alters microglia in dopaminoceptive nuclei. Br J Pharmacol. 2025. 10.1111/BPH.70081. 40400165 10.1111/bph.70081

[33] Zhang H, Largent-Milnes TM, Vanderah TW. Glial neuroimmune signaling in opioid reward. Brain Res Bull. 2020;155:102–11. 31790721 10.1016/j.brainresbull.2019.11.012 PMC6946383

[34] Hol EM, Pekny M. Glial fibrillary acidic protein (GFAP) and the astrocyte intermediate filament system in diseases of the central nervous system. Curr Opin Cell Biol. 2015;32:121–30. 25726916 10.1016/j.ceb.2015.02.004

[35] de Haas AH, Boddeke HWGM, Biber K. Region-specific expression of immunoregulatory proteins on microglia in the healthy CNS. Glia. 2008;56:888–94. 18338796 10.1002/glia.20663

[36] Clark DPQ, Perreau VM, Shultz SR, Brady RD, Lei E, Dixit S, et al. Inflammation in traumatic brain injury: roles for toxic A1 astrocytes and microglial-astrocytic crosstalk. Neurochem Res. 2019;44:1410–24. 30661228 10.1007/s11064-019-02721-8

[37] Raghavendra V, Tanga FY, DeLeo JA. Complete Freunds adjuvant-induced peripheral inflammation evokes glial activation and proinflammatory cytokine expression in the CNS. Eur J Neurosci. 2004;20:467–73. 15233755 10.1111/j.1460-9568.2004.03514.x

[38] Kaushik DK, Gupta M, Das S, Basu A. Krüppel-like factor 4, a novel transcription factor regulates microglial activation and subsequent neuroinflammation. J Neuroinflammation. 2010;7:68. 20946687 10.1186/1742-2094-7-68 PMC2965135

[39] Myers KM, Bechtholt-Gompf AJ, Coleman BR, Carlezon WA. Extinction of conditioned opiate withdrawal in rats in a two-chambered place conditioning apparatus. Nat Protoc. 2012;7:517–26. 22362157 10.1038/nprot.2011.458 PMC4957553

[40] Schulteis G, Markou A, Gold LH, Stinus L, Koob GF. Relative sensitivity to naloxone of multiple indices of opiate withdrawal: a quantitative dose-response analysis. J Pharmacol Exp Ther. 1994;271:1391–8. 7996451

[41] Pintér-Kübler B, Ferenczi S, Núnez C, Zelei E, Polyák Á, Milanés MV, et al. Differential changes in expression of stress- and metabolic-related neuropeptides in the rat hypothalamus during morphine dependence and withdrawal. PLoS ONE. 2013;8:1–12. 10.1371/journal.pone.0067027 PMC3689674 23805290

[42] García-Pérez D, Ferenczi S, Kovács KJ, Laorden ML, Milanés MV, Núñez C. Different contribution of glucocorticoids in the basolateral amygdala to the formation and expression of opiate withdrawal-associated memories. Psychoneuroendocrinology. 2016;74:350–62. 27728875 10.1016/j.psyneuen.2016.09.020

[43] Ferenczi S, Núñez C, Pintér-Kübler B, Földes A, Martín F, Márkus VL, et al. Changes in metabolic-related variables during chronic morphine treatment. Neurochem Int. 2010;57:323–30. 20600437 10.1016/j.neuint.2010.06.011

[44] Frenois F, Cador M, Caillé S, Stinus L, Le Moine C. Neural correlates of the motivational and somatic components of naloxone-precipitated morphine withdrawal. Eur J Neurosci. 2002;16:1377–89. 12405997 10.1046/j.1460-9568.2002.02187.x

[45] Myers KM, Carlezon WA. D-cycloserine facilitates extinction of naloxone-induced conditioned place aversion in morphine-dependent rats. Biol Psychiatry. 2010;67:85–7. 19782965 10.1016/j.biopsych.2009.08.015 PMC4961034

[46] Ye J, Coulouris G, Zaretskaya I, Cutcutache I, Rozen S, Madden TL. Primer-BLAST: a tool to design target-specific primers for polymerase chain reaction. BMC Bioinformatics. 2012;13:134. 22708584 10.1186/1471-2105-13-134 PMC3412702

[47] Vandesompele J, De Preter K, Pattyn F, Poppe B, Van Roy N, De Paepe A, et al. Accurate normalization of real-time quantitative RT-PCR data by geometric averaging of multiple internal control genes. Genome Biol. 2002;3:RESEARCH0034. 12184808 10.1186/gb-2002-3-7-research0034 PMC126239

[48] Benavides M, Laorden ML, García-Borrón JC, Milanés MV. Regulation of tyrosine hydroxylase levels and activity and Fos expression during opioid withdrawal in the hypothalamic PVN and medulla oblongata catecholaminergic cell groups innervating the PVN. Eur J Neurosci. 2003;17:103–12. 12534973 10.1046/j.1460-9568.2003.02434.x

[49] Navarro-Zaragoza J, Núñez C, Ruiz-Medina J, Laorden ML, Valverde O, Milanés MV. CRF₂ mediates the increased noradrenergic activity in the hypothalamic paraventricular nucleus and the negative state of morphine withdrawal in rats. Br J Pharmacol. 2011;162:851–62. 20973778 10.1111/j.1476-5381.2010.01090.x PMC3042196

[50] Taylor JR, Olausson P, Quinn JJ, Torregrossa MM. Targeting extinction and reconsolidation mechanisms to combat the impact of drug cues on addiction. Neuropharmacology. 2009;56(Suppl 1):186–95. 18708077 10.1016/j.neuropharm.2008.07.027 PMC2635342

[51] Kalivas PW, O’Brien C. Drug addiction as a pathology of staged neuroplasticity. Neuropsychopharmacology. 2008;33:166–80. 17805308 10.1038/sj.npp.1301564

[52] Haydon PG, Blendy J, Moss SJ, Rob Jackson F. Astrocytic control of synaptic transmission and plasticity: a target for drugs of abuse? Neuropharmacology. 2009;56:83–90. 10.1016/j.neuropharm.2008.06.050. 18647612 10.1016/j.neuropharm.2008.06.050 PMC2636575

[53] Cornell J, Salinas S, Huang HY, Zhou M. Microglia regulation of synaptic plasticity and learning and memory. Neural Regen Res. 2022;17:705–16. 10.4103/1673-5374.322423. 34472455 10.4103/1673-5374.322423 PMC8530121

[54] Wikler A. Dynamics of drug dependence. Arch Gen Psychiatry. 1973;28:611. 4700675 10.1001/archpsyc.1973.01750350005001

[55] Franco-García A, Fernández-Gómez FJ, Gómez-Murcia V, Hidalgo JM, Milanés MV, Núñez C. Molecular mechanisms underlying the retrieval and extinction of morphine withdrawal-associated memories in the basolateral amygdala and dentate gyrus. Biomedicines. 2022;10:588. 35327388 10.3390/biomedicines10030588 PMC8945324

[56] Franco-García A, Gómez-Murcia V, Fernández-Gómez FJ, González-Andreu R, Hidalgo JM, Victoria Milanés M, et al. Morphine-withdrawal aversive memories and their extinction modulate H4K5 acetylation and Brd4 activation in the rat hippocampus and basolateral amygdala. Biomed Pharmacother. 2023;165:115055. 37356373 10.1016/j.biopha.2023.115055

[57] Alonso E, Garrido E, Díez-Fernández C, Pérez-García C, Herradón G, Ezquerra L, et al. Yohimbine prevents morphine-induced changes of glial fibrillary acidic protein in brainstem and α2-adrenoceptor gene expression in hippocampus. Neurosci Lett. 2007;412:163–7. 17123717 10.1016/j.neulet.2006.11.002

[58] Lazriev IL, Kiknadze GI, Kutateladze II, Nebieridze MI. Regulatory Mechanisms of Metabolic Processes in Brain. Byulleten Eksperimental noi Biologii i Meditsiny. 2001;131.

[59] Beitner-Johnson D, Guitart X, Nestler EJ. Glial fibrillary acidic protein and the mesolimbic dopamine system: regulation by chronic morphine and Lewis-Fischer strain differences in the rat ventral tegmental area. J Neurochem. 1993;61:1766–73. 8228992 10.1111/j.1471-4159.1993.tb09814.x

[60] Hutchinson MR, Lewis SS, Coats BD, Skyba DA, Crysdale NY, Berkelhammer DL, et al. Reduction of opioid withdrawal and potentiation of acute opioid analgesia by systemic AV411 (ibudilast). Brain Behav Immun. 2009;23:240–50. 18938237 10.1016/j.bbi.2008.09.012 PMC2662518

[61] Coffey KR, Lesiak AJ, Marx RE, Vo EK, Garden GA, Neumaier JF. A cAMP-related gene network in microglia is inversely regulated by morphine tolerance and withdrawal. Biological Psychiatry Global Open Science. 2022;2:180–9. 35441155 10.1016/j.bpsgos.2021.07.011 PMC9015218

[62] Parekh SV, Adams LO, Barkell GA, Paniccia JE, Reissner KJ, Lysle DT. Dorsal hippocampal astrocytes mediate the development of heroin withdrawal-enhanced fear learning. Psychopharmacology. 2024;241:1265–75. 38396195 10.1007/s00213-024-06562-4 PMC11106136

[63] Parekh SV, Paniccia JE, Lebonville CL, Lysle DT. Dorsal hippocampal interleukin-1 signaling mediates heroin withdrawal-enhanced fear learning. Psychopharmacology. 2020;237:3653–64. 32860071 10.1007/s00213-020-05645-2 PMC7686097

[64] Yan Y, Truitt B, Tao J, Boyles SM, Antoine D, Hulme W, et al. Single-cell profiling of glial cells from the mouse amygdala under opioid dependent and withdrawal states. iScience. 2023;26:108166. 37915593 10.1016/j.isci.2023.108166 PMC10616319

[65] O’Sullivan SJ, Malahias E, Park J, Srivastava A, Reyes BAS, Gorky J, et al. Single-cell glia and neuron gene expression in the central amygdala in opioid withdrawal suggests inflammation with correlated gut dysbiosis. Front Neurosci. 2019. 10.3389/fnins.2019.00665. 31333398 10.3389/fnins.2019.00665 PMC6619439

[66] Navarro-Zaragoza J, Hidalgo JM, Laorden ML, Milanés MV. Glucocorticoid receptors participate in the opiate withdrawal-induced stimulation of rats NTS noradrenergic activity and in the somatic signs of morphine withdrawal. Br J Pharmacol. 2012;166:2136–47. 22364199 10.1111/j.1476-5381.2012.01918.x PMC3402777

[67] Martínez-Laorden E, Almela P, Milanés M-V, Laorden M-L. Expression of heat shock protein 27 and troponin T and troponin I after naloxone-precipitated morphine withdrawal. Eur J Pharmacol. 2015;766:142–50. 26452515 10.1016/j.ejphar.2015.10.006

[68] Hasanein P, Shakeri S. Pregabalin role in inhibition of morphine analgesic tolerance and physical dependency in rats. Eur J Pharmacol. 2014;742:113–7. 25220244 10.1016/j.ejphar.2014.08.030

[69] Gold LH, Stinus L, Inturrisi CE, Koob GF. Prolonged tolerance, dependence and abstinence following subcutaneous morphine pellet implantation in the rat. Eur J Pharmacol. 1994;253. 10.1016/0014-2999(94)90755-2 8013548

[70] Ngai SH, Berkowitz BA, Yang JC, Hempstead J, Spector S. Pharmacokinetics of naloxone in rats and in man. Anesthesiology. 1976;44:398–401. 1267205 10.1097/00000542-197605000-00008

[71] Jokinen V, Sidorova Y, Viisanen H, Suleymanova I, Tiilikainen H, Li Z, et al. Differential spinal and supraspinal activation of glia in a rat model of morphine tolerance. Neuroscience. 2018;375:10–24. 29421434 10.1016/j.neuroscience.2018.01.048

[72] Sofroniew MV. Molecular dissection of reactive astrogliosis and glial scar formation. Trends Neurosci. 2009;32:638–47. 19782411 10.1016/j.tins.2009.08.002 PMC2787735

[73] Bettcher BM, Olson KE, Carlson NE, McConnell BV, Boyd T, Adame V, et al. Astrogliosis and episodic memory in late life: higher GFAP is related to worse memory and white matter microstructure in healthy aging and Alzheimer’s disease. Neurobiol Aging. 2021;103:68–77. 33845398 10.1016/j.neurobiolaging.2021.02.012 PMC8313091

[74] Miyashita T, Murakami K, Kikuchi E, Ofusa K, Mikami K, Endo K, et al. Glia transmit negative valence information during aversive learning in *Drosophila*. Science. 2023;382:eadf7429. 38127757 10.1126/science.adf7429

[75] Lei Z, Xie L, Li CH, Lam YY, Ramkrishnan AS, Fu Z, et al. Chemogenetic activation of astrocytes in the basolateral amygdala contributes to fear memory formation by modulating the amygdala–prefrontal cortex communication. Int J Mol Sci. 2022;23:6092. 35682767 10.3390/ijms23116092 PMC9181030

[76] Adamsky A, Kol A, Kreisel T, Doron A, Ozeri-Engelhard N, Melcer T, et al. Astrocytic activation generates de novo neuronal potentiation and memory enhancement. Cell. 2018;174:59-71.e14. 29804835 10.1016/j.cell.2018.05.002

