# Supplementary material for: Regulation of glial markers expression in the rat basolateral amygdala and hippocampus during morphine aversive memory retrieval and its extinction
Source: Behav Brain Funct. 2025 Dec 14;22:4. doi: 10.1186/s12993-025-00313-x (PMC12821304; doi:10.1186/s12993-025-00313-x)
Supplement: Supplementary file 1 — Supplementary Material 1. [file 12993_2025_313_MOESM1_ESM.docx]

| **Table S1. Statistical analyses.** | | | | | | | |
| --- | --- | --- | --- | --- | --- | --- | --- |
| Brain nucleus (if applicable) | Parameter | Groups | Analysis | *F*actors | Result | | Related *F*igure in manuscript |
| NA | Area under the curve from weight gain data | PLA vs. MOR | Unpaired t-test | Pharmacological treatment | *t*_(62)_ = 4.619; P < 0.001 | | 1C |
|  | Weight gain in paired chambers (g) | PLA-Sal vs. PLA-Nx  MOR-Sal vs. MOR-Nx | RM Two-way ANOVA + Hom-Sidak post-hoc test | Naloxone or saline injection | *F* _(1, 128)_= 2.547; P = 0.1130 | PLA-Sal vs. PLA-Nx; P = 0.0007  MOR-Sal vs. MOR-Nx; P = 0.1229 | 1D |
|  |  |  |  | Morphine or placebo pellets | *F* _(1, 128)_= 8.070; P = 0.0052 |  |  |
|  |  |  |  | Interaction | *F* _(1, 128)_= 13.99; P = 0.0003 |  |  |
|  |  |  |  | Subject | *F* _(128, 128)_= 1.165; P = 0.1947 |  |  |
|  | Preference Score (s) | PLA-CPA test vs. MOR-CPA test  (Experiment 1) | Unpaired t-test | Pharmacological treatment | *t*_(21)_ = 4.356; P < 0.001 | | 2A |
|  | Preference Score (entries) |  |  |  | *t*_(21)_ = 2.098; P = 0.0482 | | 2B |
|  | Time in paired chambers (s) | PLA-Pretest vs. PLA-CPA test  MOR-Pretest vs. MOR-CPA test  (Experiment 1) | RM Three-way ANOVA + Holm-Sidak post-hoc test | Test | *F*_(1, 21)_ = 2.382; P = 0.1377 | MOR-Pretest (Nx) vs. MOR-CPA test (Nx); P = 0.0035  MOR-Pretest (Sal) vs. MOR-CPA test (Sal); P = 0.0375  MOR-CPA test (Nx) vs. MOR-CPA test (Sal); P < 0.0001 | 2C |
|  |  |  |  | Naloxone or saline injection | *F*_(1, 21)_ = 15.43; P < 0.001 |  |  |
|  |  |  |  | Morphine or placebo pellets | *F*_(1, 21)_ = 0.8778; P = 0.3595 |  |  |
|  |  |  |  | Interaction 1: test x naloxone or saline injection | *F*_(1, 21)_ = 18.98; P < 0.001 |  |  |
|  |  |  |  | Interaction 2: test x morphine or placebo pellets | *F*_(1, 21)_ = 0.1339; P = 0.7181 |  |  |
|  |  |  |  | Interaction 3: naloxone or saline injection x morphine or placebo pellets | *F*_(1, 21)_ = 1.958; P = 0.1763 |  |  |
|  |  |  |  | Interaction 4: test x naloxone or saline injection x morphine or placebo pellets | *F*_(1, 21)_ = 7.426; P < 0.0127 |  |  |
|  | Entries to paired chamber |  |  | Test | *F* _(1, 21)_ = 0.5647; P = 0.4607 | Multiple comparisons not significant | 2D |
|  |  |  |  | Naloxone or saline injection | *F* _(1, 21)_ = 3.347; P = 0.0816 |  |  |
|  |  |  |  | Morphine or placebo pellets | *F* _(1, 21)_ = 0.5765; P = 0.4561 |  |  |
|  |  |  |  | Interaction 1: test x naloxone or saline injection | *F* _(21, 21)_ = 1.470; P = 0.2388 |  |  |
|  |  |  |  | Interaction 2: test x morphine or placebo pellets | *F* _(1, 21)_ = 6.737; P = 0.0169 |  |  |
|  |  |  |  | Interaction 3: naloxone or saline injection x morphine or placebo pellets | *F*_(1, 21)_= 0.2940; P = 0.5934 |  |  |
|  |  |  |  | Interaction 4: test x naloxone or saline injection x morphine or placebo pellets | *F* _(1, 21)_= 4.400; P = 0.0482 |  |  |
|  | Preference Score (s) | PLA-CPA test vs. MOR-CPA vs. PLA-Ext test vs. MOR-Ext test (Experiment 2) | RM Two-way ANOVA + Holm-Sidak post-hoc test | Pharmacological treatment | *F* _(1, 40)_= 11.04; P = 0.0019 | PLA-CPA test vs. PLA-Ext test; P = 0.3634  MOR-CPA test vs. MOR-Ext test; P = 0.0016  PLA-CPA test vs MOR-CPA test; P = 0.0007  PLA-Ext test vs MOR-Ext test; P = 0.0872 | 2E |
|  |  |  |  | Test | *F* _(1, 40)_= 9.771; P = 0.0033 |  |  |
|  |  |  |  | Interaction | *F* _(1, 40)_= 3.116; P = 0.0852 |  |  |
|  |  |  |  | Subject | *F* _(40, 40)_= 2.098; P = 0.0106 |  |  |
|  | Preference Score (entries) | PLA-CPA test vs. MOR-CPA vs. PLA-Ext test vs. MOR-Ext test (Experiment 2) | RM Two-way ANOVA + Holm-Sidak post-hoc test | Pharmacological treatment | *F* _(1, 38)_= 7.993; P = 0.0075 | PLA-CPA test vs. PLA-Ext test; P = 0.3109  MOR-CPA test vs. MOR-Ext test; P = 0.0198  PLA-CPA test vs. MOR-CPA test; P = 0.0067  PLA-Ext test vs. MOR-Ext test; P = 0.0514 | 2F |
|  |  |  |  | Test | *F* _(1, 38)_= 6.834; P = 0.0127 |  |  |
|  |  |  |  | Interaction | *F* _(1, 38)_= 1.268; P = 0.2672 |  |  |
|  |  |  |  | Subject | *F* _(38, 38)_= 3.627; P < 0.0001 |  |  |
|  | Time in paired chamber (s) | PLA-Pretest vs. PLA-CPA test vs. PLA-Ext test  MOR-Pretest vs. MOR-CPA test vs. MOR-Ext test  (Experiment 2) | RM Three-way ANOVA + Holm-Sidak post-hoc test | Test | *F* _(2, 82)_= 8.567; P < 0.001 | MOR-Pretest (Nx) vs. MOR-CPA test (Nx); P < 0.0001  MOR-Pretest (Nx) vs. MOR-Ext test (Nx); P = 0.0002  MOR-Pretest (Sal) vs. MOR-CPA test (Sal); P < 0.0001  MOR-CPA test (Nx) vs. MOR-CPA test (Sal); P < 0.0001  MOR-CPA test (Sal) vs. MOR-Ext test (Sal); P = 0.0024  MOR-Ext test (Nx) vs. MOR-Ext test (Sal); P = 0.0366 | 2G |
|  |  |  |  | Naloxone or saline injection | *F* _(1, 41)_= 19.03; P < 0.0001 |  |  |
|  |  |  |  | Morphine or placebo pellets | *F* _(1, 41)_= 0.1781; P = 0.6752 |  |  |
|  |  |  |  | Interaction 1: test x naloxone or saline injection | *F* _(2, 82)_= 21.04; P < 0.0001 |  |  |
|  |  |  |  | Interaction 2: test x morphine or placebo pellets | *F* _(2, 82)_= 0.04112; P = 0.9597 |  |  |
|  |  |  |  | Interaction 3: naloxone or saline injection x morphine or placebo pellets | *F* _(1, 41)_= 6.713; P = 0.0132 |  |  |
|  |  |  |  | Interaction 4: test x naloxone or saline injection x morphine or placebo pellets | *F* _(2, 82)_= 6.118; P = 0.0033 |  |  |
|  | Entries to paired chamber | PLA-Pretest vs. PLA-CPA test vs. PLA-Ext test  MOR-Pretest vs. MOR-CPA test vs. MOR-Ext test  (Experiment 2) | RM Three-way ANOVA + Holm-Sidak post-hoc test | Test | *F* _(2, 76)_= 0.9101; P = 0.4068 | MOR-CPA test (Nx) vs. MOR-CPA test (Sal); P < 0.0001 | 2H |
|  |  |  |  | Naloxone or saline injection | *F* _(1, 38)_= 3.522; P = 0.0682 |  |  |
|  |  |  |  | Morphine or placebo pellets | *F* _(1, 38)_= 0.5574; P = 0.4599 |  |  |
|  |  |  |  | Interaction 1: test x naloxone or saline injection | *F* _(2, 76)_= 8.915; P < 0.001 |  |  |
|  |  |  |  | Interaction 2: test x morphine or placebo pellets | *F* _(2, 76)_= 6.178; P = 0.0033 |  |  |
|  |  |  |  | Interaction 3: naloxone or saline injection x morphine or placebo pellets | *F* _(1, 38)_= 5.517; P = 0.0241 |  |  |
|  |  |  |  | Interaction 4: test x naloxone or saline injection x morphine or placebo pellets | *F* _(2, 76)_= 4.948; P = 0.0095 |  |  |
| Basolateral amygdala | *gfap* mRNA levels (%) | PLA-CPA test vs. MOR-CPA test vs. PLA-Ext test vs. MOR-Ext test | Two-Way ANOVA + Holm-Sidak’s post-hoc | Pharmacological treatment | *F* _(1, 18)_ = 0.2137; P = 0.6494 | | 3A |
|  |  |  |  | Behaviour | *F* _(1, 18)_ = 3.419; P = 0.0810 | |  |
|  |  |  |  | Interaction | *F*_(1, 18)_= 0.3019; P = 0.5895 | |  |
|  |  | PLA-CPA test vs. MOR-CPA test | Unpaired t-test | Pharmacological treatment | *t*_(9)_ = 0.9443; P = 0.3697 | |  |
|  |  | PLA-Ext test vs. MOR-Ext test | Unpaired t-test | Pharmacological treatment | *t*_(9)_ = 0.05159; P = 0.9600 | |  |
|  |  | PLA-Day 6 vs. MOR-Day 6 vs. PLA-Day 10 vs. MOR-Day 10 | Two-Way ANOVA + Holm-Sidak’s post-hoc | Pharmacological treatment | *F* _(1, 15)_ = 0.2332; P = 0.6361 | | 3A’ |
|  |  |  |  | Days | *F* _(1, 15)_ = 0.1345; P = 0.7190 | |  |
|  |  |  |  | Interaction | *F* _(1, 15)_ = 0.8404; P = 0.3738 | |  |
|  |  | PLA-Day 6 va. MOR-Day 6 | Unpaired t-test | Pharmacological treatment | *t*_(5)_ = 1.042; P = 0.3453 | |  |
|  |  | PLA-Day 10 va. MOR-Day 10 | Unpaired t-test | Pharmacological treatment | *t*_(10)_ = 0.3361; P = 0.7437 | |  |
|  | *aif1* mRNA levels (%) | PLA-CPA test vs. MOR-CPA test vs. PLA-Ext test vs. MOR-Ext test | Two-Way ANOVA + Hom-Sidak’s post-hoc | Pharmacological treatment | *F* _(1, 19)_ = 1.845; P = 0.1903 | | 3B |
|  |  |  |  | Behaviour | *F* _(1, 19)_ = 4.357; P = 0.0506 | |  |
|  |  |  |  | Interaction | *F*_(1, 19)_= 0.3507; P = 0.5607 | |  |
|  |  | PLA-CPA test vs. MOR-CPA test | Unpaired t-test | Pharmacological treatment | *t*_(10)_ = 1.950; P = 0.0797 | |  |
|  |  | PLA-Ext test vs. MOR-Ext test | Unpaired t-test | Pharmacological treatment | *t*_(10)_ = 0.4282; P = 0.6786 | |  |
|  |  | PLA-Day 6 vs. MOR-Day 6 vs. PLA-Day 10 vs. MOR-Day 10 | Two-Way ANOVA + Holm-Sidak’s post-hoc | Pharmacological treatment | *F* _(1, 15)_ = 4.083; P = 0.0616 | | 3B’ |
|  |  |  |  | Days | *F* _(1, 15)_ = 0.0061; P = 0.9386 | |  |
|  |  |  |  | Interaction | *F* _(1, 15)_ = 0.9904; P = 0.9386 | |  |
|  |  | PLA-Day 6 va. MOR-Day 6 | Unpaired t-test | Pharmacological treatment | *t*_(5)_ = 1.942; P = 0.1098 | |  |
|  |  | PLA-Day 10 va. MOR-Day 10 | Unpaired t-test | Pharmacological treatment | *t*_(10)_ = 0.8392; P = 0.4209 | |  |
|  | *itgam* mRNA levels (%) | PLA-CPA test vs. MOR-CPA test vs. PLA-Ext test vs. MOR-Ext test | Two-Way ANOVA + Holm-Sidak’s post-hoc | Pharmacological treatment | *F* _(1, 19)_ = 2.364; P = 0.1406 | | 3C |
|  |  |  |  | Behaviour | *F* _(1, 19)_ = 0.1245; P = 0.7281 | |  |
|  |  |  |  | Interaction | *F*_(1, 19)_= 0.0511; P = 0.8235 | |  |
|  |  | PLA-CPA test vs. MOR-CPA test | Unpaired t-test | Pharmacological treatment | *t*_(10)_ = 0.9498; P = 0.3646 | |  |
|  |  | PLA-Ext test vs. MOR-Ext test | Unpaired t-test | Pharmacological treatment | *t*_(9)_ = 1.219; P = 0.2537 | |  |
|  |  | PLA-Day 6 vs. MOR-Day 6 vs. PLA-Day 10 vs. MOR-Day 10 | Two-Way ANOVA + Holm-Sidak’s post-hoc | Pharmacological treatment | *F* _(1, 15)_ = 1.710; P = 0.2107 | | 3C’ |
|  |  |  |  | Days | *F* _(1, 15)_ = 0.4419; P = 0.5163 | |  |
|  |  |  |  | Interaction | *F* _(1, 15)_ = 0.2322; P = 0.6369 | |  |
|  |  | PLA-Day 6 va. MOR-Day 6 | Unpaired t-test | Pharmacological treatment | *t*_(5)_ = 1.334; P = 0.2397 | |  |
|  |  | PLA-Day 10 va. MOR-Day 10 | Unpaired t-test | Pharmacological treatment | *t*_(10)_ = 0.6394; P = 0.5369 | |  |
|  | *klf4* mRNA levels (%) | PLA-CPA test vs. MOR-CPA test vs. PLA-Ext test vs. MOR-Ext test | Two-Way ANOVA + Hom-Sidak’s post-hoc | Pharmacological treatment | *F* _(1, 19)_ = 0.0400; P = 0.8435 | | 3D |
|  |  |  |  | Behaviour | *F* _(1, 19)_ = 0.0914; P = 0.7656 | |  |
|  |  |  |  | Interaction | *F*_(1, 19)_= 0.3382; P = 0.5677 | |  |
|  |  | PLA-CPA test vs. MOR-CPA test | Unpaired t-test | Pharmacological treatment | *t*_(10)_ = 0.4847; P = 0.6383 | |  |
|  |  | PLA-Ext test vs. MOR-Ext test | Unpaired t-test | Pharmacological treatment | *t*_(9)_ = 0.3419; P = 0.7403 | |  |
|  |  | PLA-Day 6 vs. MOR-Day 6 vs. PLA-Day 10 vs. MOR-Day 10 | Two-Way ANOVA + Holm-Sidak’s post-hoc | Pharmacological treatment | *F* _(1, 15)_ = 1.609; P = 0.2239 | | 3D’ |
|  |  |  |  | Days | *F* _(1, 15)_ = 0.0343; P = 0.8556 | |  |
|  |  |  |  | Interaction | *F* _(1, 15)_ = 0.1726; P = 0.6837 | |  |
|  |  | PLA-Day 6 va. MOR-Day 6 | Unpaired t-test | Pharmacological treatment | *t*_(5)_ = 1.222; P = 0.2763 | |  |
|  |  | PLA-Day 10 va. MOR-Day 10 | Unpaired t-test | Pharmacological treatment | *t*_(10)_ = 1.178; P = 0.2659 | |  |
|  | *itgam* mRNA levels (%) vs. Time in nx-paired chamber (s) | MOR-CPA | Pearson’s correlation | NA | r = -0.865; P = 0.0278 | | 3E |
|  | *klf4* mRNA levels (%) vs. Time in nx-paired chamber (s) | MOR-CPA | Pearson’s correlation | NA | r = -0.9311; P = 0.007 | | 3F |
|  | *gfap* mRNA levels (%) vs. Time in sal-paired chamber (s) | MOR-CPA | Pearson’s correlation | NA | r = -0.8167; P = 0.0473 | | 3G |
|  | *aif1* mRNA levels (%) vs. Time in sal-paired chamber (s) | MOR-EXT | Pearson’s correlation | NA | r = -0.8498; P = 0.0322 | | 3H |
| Dentate gyrus | *gfap* mRNA levels (%) | PLA-CPA test vs. MOR-CPA test vs. PLA-Ext test vs. MOR-Ext test | Two-Way ANOVA + Holm-Sidak’s post-hoc | Pharmacological treatment | *F* _(1, 18)_ = 0.2065; P = 0.6550 | | 4A |
|  |  |  |  | Behaviour | *F* _(1, 18)_ = 0.3259; P = 0.5751 | |  |
|  |  |  |  | Interaction | *F*_(1, 18)_= 5.107; P = 0.0365 | |  |
|  |  | PLA-CPA test vs. MOR-CPA test | Unpaired t-test | Pharmacological treatment | *t*_(9)_ = 1.596; P = 0.1448 | |  |
|  |  | PLA-Ext test vs. MOR-Ext test | Unpaired t-test | Pharmacological treatment | *t*_(9)_ = 1.645; P = 0.1343 | |  |
|  |  | PLA-Day 6 vs. MOR-Day 6 vs. PLA-Day 10 vs. MOR-Day 10 | Two-Way ANOVA + Holm-Sidak’s post-hoc | Pharmacological treatment | *F* _(1, 14)_ = 1.357; P = 0.2636 | | 4A’ |
|  |  |  |  | Days | *F* _(1, 14)_ = 4.566; P = 0.0507 | |  |
|  |  |  |  | Interaction | *F* _(1, 14)_ = 0.3299; P = 0.5748 | |  |
|  |  | PLA-Day 6 va. MOR-Day 6 | Unpaired t-test | Pharmacological treatment | *t*_(5)_ = 1.357; P = 0.2329 | |  |
|  |  | PLA-Day 10 va. MOR-Day 10 | Unpaired t-test | Pharmacological treatment | *t*_(9)_ = 1.138; P = 0.2845 | |  |
|  | *aif1* mRNA levels (%) | PLA-CPA test vs. MOR-CPA test vs. PLA-Ext test vs. MOR-Ext test | Two-Way ANOVA + Holm-Sidak’s post-hoc | Pharmacological treatment | *F* _(1, 17)_ = 0.02746; P = 0.8703 | | 4B |
|  |  |  |  | Behaviour | *F* _(1, 17)_ = 0.277; P = 0.6050 | |  |
|  |  |  |  | Interaction | *F*_(1, 17)_= 0.6035; P = 0.4479 | |  |
|  |  | PLA-CPA test vs. MOR-CPA test | Unpaired t-test | Pharmacological treatment | *t*_(8)_ = 0.9326; P = 0.3783 | |  |
|  |  | PLA-Ext test vs. MOR-Ext test | Unpaired t-test | Pharmacological treatment | *t*_(9)_ = 0.3665; P = 0.7225 | |  |
|  |  | PLA-Day 6 vs. MOR-Day 6 vs. PLA-Day 10 vs. MOR-Day 10 | Two-Way ANOVA + Holm-Sidak’s post-hoc | Pharmacological treatment | *F* _(1, 14)_ = 19.07; P = 0.0006 | PLA-Day 10 vs. MOR-Day 10; P < 0.0001  MOR-Day 6 vs. MOR-Day 10; P < 0.0001 | 4B’ |
|  |  |  |  | Days | *F* _(1, 14)_ = 20.35; P = 0.0005 |  |  |
|  |  |  |  | Interaction | *F* _(1, 14)_ = 22.32; P = 0.0003 |  |  |
|  |  | PLA-Day 6 va. MOR-Day 6 | Unpaired t-test | Pharmacological treatment | *t*_(5)_ = 0.6320; P = 0.5552 | |  |
|  |  | PLA-Day 10 va. MOR-Day 10 | Unpaired t-test | Pharmacological treatment | *t*_(9)_ = 6.008; P < 0.001 | |  |
|  | *itgam* mRNA levels (%) | PLA-CPA test vs. MOR-CPA test vs. PLA-Ext test vs. MOR-Ext test | Two-Way ANOVA + Holm-Sidak’s post-hoc | Pharmacological treatment | *F* _(1, 15)_ = 3.318; P = 0.0885 | | 4C |
|  |  |  |  | Behaviour | *F* _(1, 15)_ = 1.732; P = 0.2080 | |  |
|  |  |  |  | Interaction | *F*_(1, 15)_= 1.928; P = 0.1852 | |  |
|  |  | PLA-CPA test vs. MOR-CPA test | Unpaired t-test | Pharmacological treatment | *t*_(7)_ = 0.6787; P = 0.5191 | |  |
|  |  | PLA-Ext test vs. MOR-Ext test | Unpaired t-test | Pharmacological treatment | *t*_(9)_ = 1.759; P = 0.1125 | |  |
|  |  | PLA-Day 6 vs. MOR-Day 6 vs. PLA-Day 10 vs. MOR-Day 10 | Two-Way ANOVA + Holm-Sidak’s post-hoc | Pharmacological treatment | *F* _(1, 17)_ = 0.0275; P = 0.8703 | | 4C’ |
|  |  |  |  | Days | *F* _(1, 17)_ = 0.2777; P = 0.6050 | |  |
|  |  |  |  | Interaction | *F* _(1, 17)_ = 0.6035; P = 0.4479 | |  |
|  |  | PLA-Day 6 va. MOR-Day 6 | Unpaired t-test | Pharmacological treatment | *t*_(5)_ = 1.174; P = 0.2934 | |  |
|  |  | PLA-Day 10 va. MOR-Day 10 | Unpaired t-test | Pharmacological treatment | *t*_(10)_ = 2.193; P = 0.0531 | |  |
|  | *klf4* mRNA levels (%) | PLA-CPA test vs. MOR-CPA test vs. PLA-Ext test vs. MOR-Ext test | Two-Way ANOVA + Holm-Sidak’s post-hoc | Pharmacological treatment | *F* _(1, 15)_ = 4.061; P = 0.0622 | | 4D |
|  |  |  |  | Behaviour | *F* _(1, 15)_ = 0.090; P = 0.7682 | |  |
|  |  |  |  | Interaction | *F*_(1, 19)_= 2.028; P = 0.1749 | |  |
|  |  | PLA-CPA test vs. MOR-CPA test | Unpaired t-test | Pharmacological treatment | *t*_(8)_ = 0.8362; P = 0.4273 | |  |
|  |  | PLA-Ext test vs. MOR-Ext test | Unpaired t-test | Pharmacological treatment | *t*_(8)_ = 3.242; P = 0.0118 | |  |
|  |  | PLA-Day 6 vs. MOR-Day 6 vs. PLA-Day 10 vs. MOR-Day 10 | Two-Way ANOVA + Holm-Sidak’s post-hoc | Pharmacological treatment | *F* _(1, 14)_ = 0.7354; P = 0.4056 | | 4D’ |
|  |  |  |  | Days | *F* _(1, 14)_ = 2.370; P = 0.1460 | |  |
|  |  |  |  | Interaction | *F* _(1, 14)_ = 0.6618; P = 0.4295 | |  |
|  |  | PLA-Day 6 va. MOR-Day 6 | Unpaired t-test | Pharmacological treatment | *t*_(5)_ = 0.3528; P = 0.7386 | |  |
|  |  | PLA-Day 10 va. MOR-Day 10 | Unpaired t-test | Pharmacological treatment | *t*_(9)_ = 1.080; P = 0.3084 | |  |
| CA1 | *gfap* mRNA levels (%) | PLA-CPA test vs. MOR-CPA test vs. PLA-Ext test vs. MOR-Ext test | Two-Way ANOVA + Holm-Sidak’s post-hoc | Pharmacological treatment | *F* _(1, 15)_ = 0.7310; P = 0.4060 | | 4E |
|  |  |  |  | Behaviour | *F* _(1, 15)_ = 0.2137; P = 0.6505 | |  |
|  |  |  |  | Interaction | *F*_(1, 18)_= 2.849; P = 0.1121 | |  |
|  |  | PLA-CPA test vs. MOR-CPA test | Unpaired t-test | Pharmacological treatment | *t*_(9)_ = 1.568; P = 0.1512 | |  |
|  |  | PLA-Ext test vs. MOR-Ext test | Unpaired t-test | Pharmacological treatment | *t*_(6)_ = 1.330; P = 0.2318 | |  |
|  |  | PLA-Day 6 vs. MOR-Day 6 vs. PLA-Day 10 vs. MOR-Day 10 | Two-Way ANOVA + Holm-Sidak’s post-hoc | Pharmacological treatment | *F* _(1, 15)_ = 0.0477; P = 0.8301 | | 4E’ |
|  |  |  |  | Days | *F* _(1, 15)_ = 1.296; P = 0.2729 | |  |
|  |  |  |  | Interaction | *F* _(1, 15)_ = 3.196; P = 0.0940 | |  |
|  |  | PLA-Day 6 va. MOR-Day 6 | Unpaired t-test | Pharmacological treatment | *t*_(5)_ = 1.502; P = 0.1933 | |  |
|  |  | PLA-Day 10 va. MOR-Day 10 | Unpaired t-test | Pharmacological treatment | *t*_(10)_ = 1.214; P = 0.2528 | |  |
|  | *aif1* mRNA levels (%) | PLA-CPA test vs. MOR-CPA test vs. PLA-Ext test vs. MOR-Ext test | Two-Way ANOVA + Holm-Sidak’s post-hoc | Pharmacological treatment | *F* _(1, 15)_ = 4.992; P = 0.0411 | PLA-CPA test vs MOR-CPA test; P = 0.1063 | 4F |
|  |  |  |  |  |  | PLA-Ext test vs. MOR-Ext test; P = 0.7864 |  |
|  |  |  |  | Behaviour | *F* _(1, 15)_ = 0.0002; P = 0.9964 | |  |
|  |  |  |  | Interaction | *F*_(1, 15)_= 1.4400; P = 0.2488 | |  |
|  |  | PLA-CPA test vs. MOR-CPA test | Unpaired t-test | Pharmacological treatment | *t*_(9)_ = 2.275; P = 0.0489 | |  |
|  |  | PLA-Ext test vs. MOR-Ext test | Unpaired t-test | Pharmacological treatment | *t*_(6)_ = 0.9817; P = 0.3641 | |  |
|  |  | PLA-Day 6 vs. MOR-Day 6 vs. PLA-Day 10 vs. MOR-Day 10 | Two-Way ANOVA + Holm-Sidak’s post-hoc | Pharmacological treatment | *F* _(1, 15)_ = 1.952; P = 0.1827 | | 4F’ |
|  |  |  |  | Days | *F* _(1, 15)_ = 0.1572; P = 0.6973 | |  |
|  |  |  |  | Interaction | *F* _(1, 15)_ = 8.323; P = 0.0113 | |  |
|  |  | PLA-Day 6 va. MOR-Day 6 | Unpaired t-test | Pharmacological treatment | *t*_(5)_ = 4.710; P = 0.0053 | |  |
|  |  | PLA-Day 10 va. MOR-Day 10 | Unpaired t-test | Pharmacological treatment | *t*_(10)_ = 1.067; P = 0.3112 | |  |
|  | *itgam* mRNA levels (%) | PLA-CPA test vs. MOR-CPA test vs. PLA-Ext test vs. MOR-Ext test | Two-Way ANOVA + Holm-Sidak’s post-hoc | Pharmacological treatment | *F* _(1, 15)_ = 5.167; P = 0.0382 | PLA-CPA test vs MOR-CPA test; P = 0.0223 | 4G |
|  |  |  |  |  |  | PLA-Ext test vs. MOR-Ext test; P > 0.9999 |  |
|  |  |  |  | Behaviour | *F* _(1, 15)_ = 0.0430; P = 0.8384 | |  |
|  |  |  |  | Interaction | *F*_(1, 15)_= 4.773; P = 0.0452 | |  |
|  |  | PLA-CPA test vs. MOR-CPA test | Unpaired t-test | Pharmacological treatment | *t*_(9)_ = 5.033; P < 0.001 | |  |
|  |  | PLA-Ext test vs. MOR-Ext test | Unpaired t-test | Pharmacological treatment | *t*_(6)_ = 0.0432; P = 0.9669 | |  |
|  |  | PLA-Day 6 vs. MOR-Day 6 vs. PLA-Day 10 vs. MOR-Day 10 | Two-Way ANOVA + Holm-Sidak’s post-hoc | Pharmacological treatment | *F* _(1, 14)_ = 0.6599; P = 0.4302 | | 4G’ |
|  |  |  |  | Days | *F* _(1, 14)_ = 7.045; P = 0.0189 | Multiple comparisons not significant |  |
|  |  |  |  | Interaction | *F* _(1, 14)_ = 0.2073; P = 0.6559 | |  |
|  |  | PLA-Day 6 va. MOR-Day 6 | Unpaired t-test | Pharmacological treatment | *t*_(5)_ = 0.5653; P = 0.5963 | |  |
|  |  | PLA-Day 10 va. MOR-Day 10 | Unpaired t-test | Pharmacological treatment | *t*_(9)_ = 0.4443; P = 0.6673 | |  |
|  | *klf4* mRNA levels (%) | PLA-CPA test vs. MOR-CPA test vs. PLA-Ext test vs. MOR-Ext test | Two-Way ANOVA + Holm-Sidak’s post-hoc | Pharmacological treatment | *F* _(1, 15)_ = 9.709; P = 0.0071 | PLA-CPA test vs MOR-CPA test; P = 0.0064 | 4H |
|  |  |  |  |  |  | PLA-Ext test vs. MOR-Ext test; P > 0.9890 |  |
|  |  |  |  | Behaviour | *F* _(1, 15)_ = 3.354; P = 0.0870 | |  |
|  |  |  |  | Interaction | *F*_(1, 19)_= 4.561; P = 0.0496 | |  |
|  |  | PLA-CPA test vs. MOR-CPA test | Unpaired t-test | Pharmacological treatment | *t*_(9)_ = 3.290; P = 0.0094 | |  |
|  |  | PLA-Ext test vs. MOR-Ext test | Unpaired t-test | Pharmacological treatment | *t*_(6)_ = 1.314; P = 0.2369 | |  |
|  |  | PLA-Day 6 vs. MOR-Day 6 vs. PLA-Day 10 vs. MOR-Day 10 | Two-Way ANOVA + Holm-Sidak’s post-hoc | Pharmacological treatment | *F* _(1, 15)_ = 0.8969; P = 0.3586 | | 4H’ |
|  |  |  |  | Days | *F* _(1, 15)_ = 0.0066; P = 0.9365 | |  |
|  |  |  |  | Interaction | *F* _(1, 15)_ = 0.0006; P = 0.9807 | |  |
|  |  | PLA-Day 6 va. MOR-Day 6 | Unpaired t-test | Pharmacological treatment | *t*_(5)_ = 0.5713; P = 0.5925 | |  |
|  |  | PLA-Day 10 va. MOR-Day 10 | Unpaired t-test | Pharmacological treatment | *t*_(10)_ = 0.8106; P = 0.4365 | |  |
| DG | *itgam* mRNA levels (%) vs. Preference Score (s) | MOR-CPA | Pearson’s correlation | NA | r = 0.9260; P = 0.0240 | | 4I |
| CA1 | *itgam* mRNA levels (%) vs. Time in sal-paired chamber (s) | MOR-CPA | Pearson’s correlation | NA | r = -0.8962; P = 0.0156 | | 4J |
|  | *gfap* mRNA levels (%) vs. Time in sal-paired chamber (s) | MOR-CPA | Pearson’s correlation | NA | r = -0.8167; P = 0.0473 | | 4K |
|  | *klf4* mRNA levels (%) vs. Time in sal-paired chamber (s) | MOR-CPA | Pearson’s correlation | NA | r = -0.9053; P = 0.0130 | | 4L |
|  | *klf4* mRNA levels (%) vs. Time in nx-paired chamber (s) | MOR-EXT | Pearson’s correlation | NA | r = -0.9516; P = 0.0484 | | 4M |
|  | *klf4* mRNA levels (%) vs. Time in sal-paired chamber (s) | MOR-EXT | Pearson’s correlation | NA | r = 0.9518; P = 0.0482 | | 4N |
|  | *gfap* mRNA levels (%) vs. Change Score (s) | MOR-EXT | Pearson’s correlation | NA | r = -0.9811; P = 0.0189 | | 4O |
|  | *klf4* mRNA levels (%) vs. Preference Score (s) | MOR-EXT | Pearson’s correlation | NA | r = -0.9757; P = 0.0243 | | 4P |
